# RG7774 (Vicasinabin), an orally bioavailable cannabinoid receptor 2 (CB2R) agonist, decreases retinal vascular permeability, leukocyte adhesion, and ocular inflammation in animal models

**DOI:** 10.3389/fphar.2024.1426446

**Published:** 2024-07-12

**Authors:** Uwe Grether, Richard H. Foxton, Sabine Gruener, Claudia Korn, Atsushi Kimbara, Anja Osterwald, Elisabeth Zirwes, Sabine Uhles, Janina Thoele, Nadine Colé, Mark Rogers-Evans, Stephan Röver, Matthias Nettekoven, Rainer E. Martin, Jean-Michel Adam, Jürgen Fingerle, Caterina Bissantz, Wolfgang Guba, André Alker, Anna M. Szczesniak, Ross F. Porter, Tom J. Toguri, Franco Revelant, Agnès Poirier, Camille Perret, Lotte Winther, Antonello Caruso, Filomena Fezza, Mauro Maccarrone, Melanie E. M. Kelly, Sascha Fauser, Christoph Ullmer

**Affiliations:** ^1^ F. Hoffmann-La Roche Ltd, Pharma Research and Early Development, Roche Innovation Center Basel, Basel, Switzerland; ^2^ Departments of Pharmacology, Anesthesia, Ophthalmology and Visual Sciences, Dalhousie University, Halifax, NS, Canada; ^3^ Department of Experimental Medicine, University of Rome Tor Vergata, Rome, Italy; ^4^ Department of Biotechnological and Applied Clinical Sciences, University of L’Aquila, L’Aquila, Italy; ^5^ European Center for Brain Research (CERC), Santa Lucia Foundation IRCCS, Rome, Italy

**Keywords:** RG7774, CB2R agonist, LPS-induced uveitis, STZ-induced diabetic retinopathy, laser-induced CNV, leukostasis, vascular permeability, inflammation

## Abstract

**Introduction:**

Preclinical studies suggest that cannabinoid receptor type 2 (CB2R) activation has a therapeutic effect in animal models on chronic inflammation and vascular permeability, which are key pathological features of diabetic retinopathy (DR). A novel CB2R agonist, triazolopyrimidine RG7774, was generated through lead optimization of a high-throughput screening hit. The aim of this study was to characterize the pharmacology, absorption, distribution, metabolism, elimination, and toxicity (ADMET) profile of RG7774, and to explore its potential for managing the key pathological features associated with retinal disease in rodents.

**Methods:**

The *in vitro* pharmacology of RG7774 was investigated for CB2R binding and receptor activation using recombinant human and mouse CB2R expression in Chinese hamster ovary cells, and endogenous CB2R expression in human Jurkat cells, and rat and mouse spleen cells. The ADMET profile was evaluated and the effects of RG7774 on retinal permeability, leukocyte adhesion, and choroidal neovascularization (CNV) were investigated in rodent models of retinal disease. Pharmacokinetic (PK) parameters and the exposure-response relationship were characterized in healthy animals and in animals with laser-induced CNV.

**Results:**

RG7774 was found to be a potent (EC_50_: 2.8 nM and K_i_: 51.3 nM), selective, and full CB2R agonist with no signs of cannabinoid receptor type 1 (CB1R) binding or activation. The ligand showed a favorable ADMET profile and exhibited systemic and ocular exposure after oral delivery. Functional potency *in vitro* translated from recombinant to endogenous expression systems. *In vivo*, orally administered RG7774 reduced retinal permeability and leukocyte adhesion in rodents with lipopolysaccharide (LPS)-induced uveitis and streptozotocin (STZ)-induced DR, and reduced lesion areas in rats with laser-induced CNV with an ED_50_ of 0.32 mg/kg. Anatomically, RG7774 reduced the migration of retinal microglia to retinal lesions.

**Discussion:**

RG7774 is a novel, highly selective, and orally bioavailable CB2R agonist, with an acceptable systemic and ocular PK profile, and beneficial effects on retinal vascular permeability, leukocyte adhesion, and ocular inflammation in rodent animal models. Results support the development of RG7774 as a potential treatment for retinal diseases with similar pathophysiologies as addressed by the animal models.

## 1 Introduction

Diabetic retinopathy (DR) is the most common microvascular complication of type 1 and 2 diabetes and a leading cause of vision loss in adults (Teo et al., 2021). In 2020, the number of adults worldwide with DR and vision-threatening DR was estimated to be 103.1 and 28.5 million, respectively, with numbers projected to rise to 160.5 and 44.8 million, respectively, by 2025 (Teo et al., 2021). DR can be broadly classified into 2 stages: nonproliferative diabetic retinopathy (NPDR) and proliferative diabetic retinopathy (PDR) (Kusuhara et al., 2018; Simó and Hernández, 2022). In NPDR, chronic hyperglycemia which affects approximately one-third of the population with diabetes (Yau et al., 2012) causes oxidative stress, inflammation, leukocyte migration, and adherence to the capillary endothelium (leukostasis) (Kusuhara et al., 2018). This can lead to increased vascular permeability, activation of retinal microglia with subsequent neurotoxicity, and destruction of the blood-retinal barrier. In PDR, retinal hypoxia resulting from vessel occlusion induces extraretinal neovascularization accompanied by fibrovascular membrane formation (Kusuhara et al., 2018). First-line treatment for severe NPDR includes regular intravitreal (IVT) injections with anti-vascular endothelial growth factor (anti-VEGF) therapies (Adamis et al., 2020; Simó and Hernández, 2022). Although early treatment of NPDR with anti-VEGF therapies can significantly reduce the incidence of PDR and subsequent blindness (Nguyen et al., 2023), treatment is typically only initiated once visual impairment has developed (Review of Ophthalmology, 2023). This is likely because anti-VEGF injections are associated with high levels of treatment burden, patient-reported anxiety, and an infrequent but serious risk of intraocular inflammation (Senra et al., 2017; Review of Ophthalmology, 2023). Compared with IVT, noninvasive oral therapies may allow greater treatment uptake with earlier treatment, improved compliance, and, since DR is frequently bilateral, may benefit both eyes simultaneously.

Components of the endocannabinoid system (ECS), including the cannabinoid receptor types 1 and 2 (CB1R and CB2R), have been investigated as potential treatments for retinal diseases and neurodegenerative diseases due to their effects on vascular permeability, inflammation, and neuroprotection (Cabral and Griffin-Thomas, 2008; Pacher and Mechoulam, 2011; Ramirez et al., 2012; Tsujikawa and Ogura, 2012; Pacher and Kunos, 2013; Rom et al., 2013; Tarr et al., 2013; Fujii et al., 2014; Toguri et al., 2014; Eshaq et al., 2017; Rapino et al., 2018; Porter et al., 2019; Stark et al., 2021; Tang et al., 2021; Spyridakos et al., 2022; Maccarrone et al., 2023; Ontko et al., 2023). CB1R and CB2R are closely related members of the superfamily of G-protein coupled receptors (GPCR) (Howlett et al., 2002; Rapino et al., 2018; Tang et al., 2021), with 44% sequence homology in their ligand binding domains (Matsuda et al., 1990). CB1R is abundant in the central nervous system (CNS) and peripheral neurons, where it contributes to neurotransmitter release and neuroprotection (Herkenham et al., 1990; Piomelli, 2003; Pacher and Kunos, 2013; Rapino et al., 2018). CB2R is preferentially expressed on immune cells (including retinal microglia), where it plays a key role in the regulation of inflammatory mediators (Lynn and Herkenham, 1994; Cabral and Griffin-Thomas, 2008; Borowska-Fielding et al., 2018; Rapino et al., 2018; Tang et al., 2021). Preclinical studies show that the loss of CB2R exacerbates intraocular inflammation in rodent models of retinal disease (Porter et al., 2019), whereas CB2R activation reduces vascular permeability in the brain (Ramirez et al., 2012; Rom et al., 2013; Fujii et al., 2014) and retina (Stark et al., 2021; Spyridakos et al., 2022; Ontko et al., 2023), inhibits leukostasis in multiple tissues (including the liver, kidney, brain, heart (Pacher and Mechoulam, 2011), retina (Tsujikawa and Ogura, 2012; Tarr et al., 2013; Eshaq et al., 2017; Porter et al., 2019; Stark et al., 2021; Ontko et al., 2023), and iris (Toguri et al., 2014)), and modulates immune cell functions by attenuating inflammation, oxidative stress, and cell death (Horváth et al., 2012; Turcotte et al., 2016). This suggests that CB2R agonists might be suitable targets for the treatment of DR and other retinal diseases with similar pathophysiology. Unlike CB2R, CB1R activation is associated with psychotropic effects, making it unsuitable for clinical use (Pacher and Kunos, 2013; Dhopeshwarkar and Mackie, 2014; Tang et al., 2021).

A novel CB2R agonist, (*S*)-1-(5-*tert*-butyl-3-[(1-methyl-1*H*-tetrazol-5-yl)methyl]-3*H*-[1,2,3]triazolo[4,5-d]pyrimidin-7-yl)pyrrolidin-3-ol)triazolopyrimidine (RG7774), was generated in-house through lead optimization of a high-throughput screening hit (Adam et al., 2013; Nettekoven et al., 2016). The aim of this study was to characterize the *in vitro* pharmacology and the absorption, distribution, metabolism, elimination, and toxicity (ADMET) profile of RG7774, and to demonstrate by predicting *in vivo* efficacious doses upon oral administration its therapeutic potential for managing the key pathological features associated with DR in rodent models of retinal diseases, including vascular permeability, leukocyte adhesion, and inflammation.

## 2 Materials and methods

### 2.1 Synthesis and molecular docking of RG7774

#### 2.1.1 Synthesis of (*S*)-1-(5-*tert*-butyl-3-[(1-methyl-1*H*-tetrazol-5-yl)methyl]-3*H*-[1,2,3]triazolo[4,5-d]pyrimidin-7-yl)pyrrolidin-3-ol RG7774 (CAS number 1433361-02-4)

**FIGURE 1:**
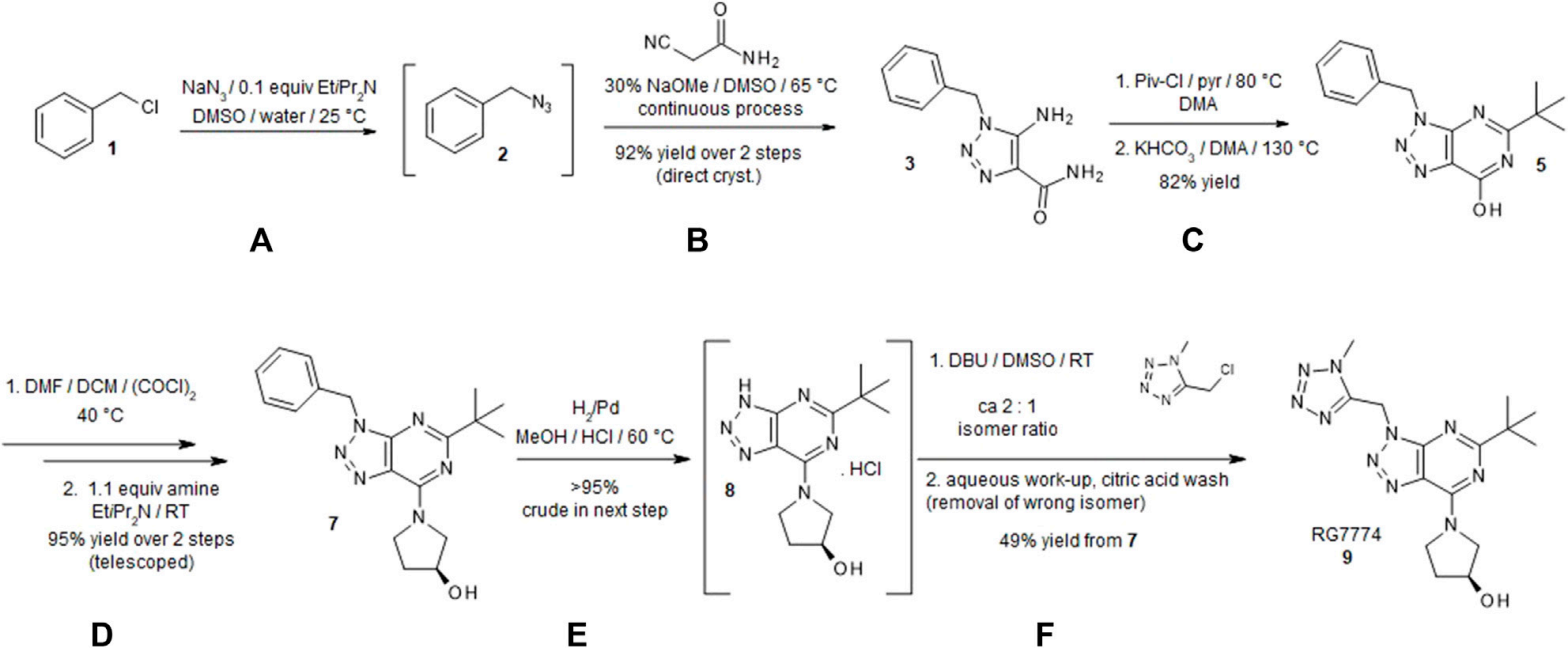
Synthesis of (*S*)-1-(5-*tert*-butyl-3-[(1-methyl-1*H*-tetrazol-5-yl)methyl]-3*H*-[1,2,3]triazolo[4,5-d]pyrimidin-7-yl)pyrrolidin-3-ol (RG7774). A detailed description of the synthesis of RG7774 can be found in Supplementary Methods S1. Briefly, the process was carried out over 8 steps as follows: **(A)** Benzyl chloride **1** was converted to azide **2**. **(B)** Under basic conditions 2-cyanoacetamide was continuously added to a solution of benzyl azide to form triazole **3** (95% yield). **(C)** Subsequent acylation with pivaloyl chloride led to the formation of 1-benzyl-5-(2,2-dimethylpropanoylamino)triazole-4-carboxamide **4**. The addition of potassium hydrogen carbonate while heating to 130°C led to ring closure and the formation of pyrimidinone **5**. **(D)** The addition of oxalyl chloride converted hydroxy-substituted triazolopyrimidine to chlorine-substituted triazolopyrimidine **6** and a nucleophilic aromatic substitution using (*S*)-pyrrolidin-3-ol led to the formation of high-yield (88%) triazolopyrimidine **7**. **(E)** The benzyl protection group on triazolopyrimidine was removed via catalytic hydrogenation to prepare for the installation of the tetrazole moiety. **(F)** Deprotected triazole **8** was alkylated with 5-(chloromethyl)-1-methyl-1*H*-tetrazole to form a mixture of 1-substituted triazolopyrimidine RG7774 and its 2-substituted regioisomer in a ratio of 2:1. After removal of the 2-substituted regioisomer using a citric acid wash, highly pure RG7774 **9** (35% yield) was obtained through crystallization. NaN_3_: Sodium azide; EtiPr_2_N: *N,N*-Diisopropylethylamine; DMSO: Dimethylsulfoxide; NaOMe: sodium methoxide; Piv-Cl: Pivaloyl chloride; pyr: Pyridine; DMA: *N,N*-Dimethylacetamide; KHCO_3_: Potassium bicarbonate; DMF: *N,N*-Dimethylformamide; DCM: Dichloromethane; (COCl)_2_: Oxalyl chloride; H_2_: Hyrodgen; Pd: Palladium; MeOH: Methanol; HCl: Hydrochloride; DBU: 1,8- Diazabicyclo[5.4.0]undec-7-ene; RT: room temperature.

Triazolopyrimidine RG7774 was synthesized starting from benzyl chloride (Adam et al., 2013) as described in Figure 1 with further details provided in Supplementary Methods S1. All chemicals were purchased from commercial sources and used without additional purification. Reactions were magnetically and mechanically stirred and monitored using either thin-layer chromatography silica gel 60 F254 TLC glass plates (Merck Millipore, Darmstadt, Germany) with visualization via ultraviolet [UV] fluorescence at 254 nm) or analytical high-performance liquid chromatography-mass spectroscopy (HPLC-MS) (Agilent 1100, Finnigan single Quadrupole ESI, autosampler Gilson 215, Chromeleon 6.7, rapid resolution cartridge Zorbax XDB 3.5 µM [21 mm × 30 mm]). The purity of new compounds determined by proton nuclear magnetic resonance (^1^H-NMR) was ≥ 95%. Flash column chromatography was performed using silica cartridges (SiliCycle Inc., Quebec, Canada), eluting with distilled technical-grade solvents on a CombiFlash RF (Teledyne ISCO, NE, United States). Preparative HPLC purification was conducted using a reverse-phase column (Phenomenex Gemini 5 mm NX-C18, 75 × 30 mm, AXIA), eluting with an acetonitrile, water, and formic acid gradient. ^1^H NMR data were recorded on a Bruker spectrometer (300, 400, 600 MHz). Chemical shifts (d) are reported in parts per million (ppm) with TMS as an internal standard. Data are reported as s = singlet, d = doublet, t = triplet, m = multiplet, br = broad signal, coupling constant(s) *J* in Hz. Service measurements were performed by the NMR or MS service teams at F. Hoffmann-La Roche Ltd., Basel, Switzerland. Characterization details for compounds **3, 5, 6, 7,** and **9** are shown below.

##### 2.1.1.1 5-Amino-1-benzyl-1*H*-1,2,3-triazole-4-carboxamide (compound 3)

High-resolution mass spectrometry (HRMS): (m/z) [M + H]^+^ defined for C_10_H_11_N_5_O: 217.0964, found: 218.1051; ^1^H NMR (600 MHz, dimethylsulfoxide- [DMSO-] *d6*) δ ppm 5.41 (s, 2 H), 6.38 (s, 2 H), 7.08 (br s, 1 H), 7.21 (d, *J* = 7.46 Hz, 2 H), 7.28–7.31 (m, 1 H), 7.34–7.37 (m, 2 H), 7.44 (br s, 1 H).

##### 2.1.1.2 3-Benzyl-5-*tert*-Butyl-4*H*-triazolo[4,5-d]pyrimidin-7-one (compound 5)

HRMS: (m/z) [M + H]^+^ calculated for C_15_H_17_N_5_O: 283.1433, found: 284.1519; ^1^H NMR (600 MHz, DMSO-*d6*) δ ppm 1.36 (s, 9 H), 5.70 (s, 2 H), 7.31–7.41 (m, 5 H), 12.18 (br s, 1 H).

##### 2.1.1.3 (3*S*)-1-(3-benzyl-5-*tert*-Butyl-triazolo[4,5-d]pyrimidin-7-yl)pyrrolidin-3-ol 3-benzyl-5-tert-butyl-7-chloro-3*H*-[1,2,3]triazolo[4,5-d]pyrimidine (compound 6)

HRMS: (m/z) [M + H]^+^ calculated for C_19_H_24_N_6_O: 352.2012, found: 353.2088; ^1^H NMR (600 MHz, DMSO-*d6*) δ ppm 1.35 (s, 9 H), 1.9–2.2 (m, 2 H), 3.7–4.2 (m, 2 H), 3.7–4.3 (m, 2 H), 4.4–4.5 (m, 1 H), 5.06 (br s, 1 H), 5.72 (s, 2 H), 7.3–7.3 (m, 1 H), 7.3–7.4 (m, 4 H); ^13^C NMR (151 MHz, DMSO-*d6*): δ = 29.5, 32.9, 39.1, 46.0, 49.2, 56.1, 68.6, 123.3, 128.0, 128.0, 128.6, 135.9, 150.1, 151.9, 174.7.

##### 2.1.1.4 (3*S*)-1-(5-*tert*-butyl-3*H*-triazolo[4,5-d]pyrimidin-7-yl)pyrrolidin-3-ol x 0.5 HCl (compound 7)

HRMS: (m/z) [M + H]^+^ calculated for C_12_H_18_N_6_O: 262.1542, found: 263.1616; ^1^H NMR (600 MHz, DMSO-*d6*) δ ppm 1.36 (s, 9 H), 1.9–2.2 (m, 2 H), 3.7–4.2 (m, 2 H), 3.7–4.3 (m, 2 H), 4.4–4.5 (m, 1 H).

##### 2.1.1.5 ((*S*)-1-(5-*tert*-Butyl-3-[(1-methyl-1*H*-tetrazol-5-yl)methyl]-3*H*-[1,2,3]triazolo[4,5-d]pyrimidin-7-yl)pyrrolidin-3-ol) RG7774 (compound 9)

HRMS: (m/z) [M + H]^+^ calculated for C_15_H_22_N_10_O: 358.1978, found: 359.2049; ^1^H NMR (600 MHz, DMSO-*d6*) δ ppm 1.35 (s, 9 H), 1.89–2.18 (m, 2 H), 3.66–3.90 (m, 2 H), 4.01–4.14 (m, 2 H), 4.17–4.20 (m, 3 H), 4.23–4.29 (m, 1 H), 4.40–4.54 (m, 1 H), 4.98–5.24 (m, 1 H), 6.14–6.18 (m, 2 H); ^13^C NMR (151 MHz, DMSO-*d6*): δ = 29.2, 31.7, 33.6, 33.8, 37.8, 38.9, 39.8, 44.9, 56.8, 67.5, 69.2, 122.7, 122.8, 149.9, 150.0, 150.6, 151.6, 151.6, 174.8.

#### 2.1.2 X-ray crystal structure and absolute configuration of RG7774

RG7774 crystals were grown by vapor diffusion using dichloromethane and a single crystal was mounted in a loop. Data were collected at room temperature (RT) on a Synergy-S diffractometer (Rigaku, Tokyo, Japan) with Cu-K-alpha-radiation (1.54184Å) and processed with the CrysAlis-package. Structure solution and refinement was performed using the ShelXTL software (Bruker AXS, Karlsruhe, Germany). Supplementary crystallographic data can be obtained free of charge from the Cambridge Crystallographic Data Centre (CCDC 22814444) via www.ccdc.cam.ac.uk/structures. Further information on data collection and structure refinement is shown in Supplementary Table S1.

#### 2.1.3 Molecular docking

The previously reported X-ray crystal structure for CB2R complexed with the CB2R agonist, AM12033 (PDB: 6KPF) (Hua et al., 2020), was used as a template for RG7774 docking. Docking experiments were performed interactively using MOE software (Chemical Computing Group, Montreal, Canada) with default settings (Molecular Operating Environment MOE, 2022). The most reasonable docking pose with respect to molecular interactions and internal conformational strain was energy-minimized within the binding pocket. Adjacent amino acid side chains were energy-minimized without restraints. The resulting docking pose was checked for consistency with the available structure-activity relationship information.

### 2.2 *In vitro* binding and functional cellular assays

#### 2.2.1 Radioligand binding assays

Competition and saturation binding assays were performed using the radiolabeled CB1R/CB2R agonist [^3^H]-CP55940 (PerkinElmer, Waltham, MA, United States) in recombinant cell systems, and the newly developed CB2R-selective agonist [^3^H]-RO6753361 (Chicca et al., 2024) in cells expressing endogenous CB1R/CB2R. Competition assays were conducted by incubating 5, 40, or 40 µg of membrane protein from CHO cells, Jurkat cells or spleen cells, respectively, with 1.5 nM [^3^H]-CP55940 or 1.5 nM [^3^H]-RO6753361 in the presence or absence of increasing concentrations of ligand (RG7774 or control) for 2 h at 30°C in a final volume of 0.2 mL of assay buffer (50 mmol/L Tris-HCl, 5 mmol/L MgCl_2_, 2.5 mmol/L EDTA, and 0.5% fatty acid-free BSA [pH 7.4] and 1% DMSO), with gentle shaking. Saturation binding assays were conducted by incubating 1–3 μg, 40 μg, or 30–40 μg of membrane protein from CHO cells, Jurkat cells, or spleen tissue, respectively, with 12 concentrations in the range of 80–0.039 nM [^3^H]-CP55940 or [^3^H]-RO6753361 for 2 h at 30°C in a final volume of 0.2 mL/well of assay buffer without DMSO. Using the control ligand, CP55940 (10 μM) to define nonspecific binding, > 95% of the total binding signal was found to be specific. Binding reactions for both assays were terminated by vacuum filtration onto 0.5% polyethylenimine presoaked GF/B filter plates (Packard BioSciences, Meriden, CT, United States) using a Filtermate cell harvester followed by 6 brief washes with 0.3 mL/well of ice-cold wash buffer. Wash buffer for CHO and Jurkat membranes comprised 50 mmol/L Tris-HCl, 5 mmol/L MgCl_2_, 2.5 mmol/L EDTA, and 0.5% fatty acid-free BSA, pH 7.4, whereas wash buffer for spleen membranes comprised 50 mmol/L Tris-HCl and 0.05% fatty acid-free BSA. Plates were dried at 50°C for 1 h and liquid scintillation counting was used to determine levels of bound radiolabel. IC_50_ values and Hill slopes were determined with a 4-parameter logistic model using ActivityBase (ID Business Solution, Guilford, United Kingdom) and pKi values were determined using the Cheng-Prusoff equation (Cheng and Prusoff, 1973).

#### 2.2.2 Cyclic adenosine monophosphate (cAMP) assays

The inhibitory effects of RG7774 on forskolin-stimulated accumulation of cAMP were determined using the cAMP-Nano-TRF detection kit (Roche Diagnostics, Penzberg, Germany), as previously described (Porter et al., 2019). CHOK1hCB1_bgal, CHOK1mCB2_bgal, and CHOK1hCB2_bgal cells (DiscoveRx, Fremont, CA, United States) were seeded 17–24 h prior to the experiment at a density of 3 × 10^4^ cells per well in a black 96-well plate with a flat, clear bottom (Corning, Wiesbaden, Germany) prior to incubating in 5% CO_2_ at 37°C in a humidified incubator. The growth medium was exchanged with Krebs Ringer bicarbonate buffer containing 1 mM IBMX and 0.1% fatty acid-free BSA and incubated at 30°C for 1 h. RG7774 or positive control (CP55940) (1 µM) was added to a 0.1 mL final assay volume and the mixture was incubated at 30°C for 30 min. The assay was stopped by adding 50 μL 3x lysis reagent and shaking for 2 h at RT. The time-resolved energy transfer was measured using an LF502 Nanoscan FLT (IOM, Berlin, Germany) equipped with a laser excitation source. cAMP content was determined from a standard curve spanning 10–0.13 nM cAMP. Efficacies were expressed as a percentage relative to the positive control.

#### 2.2.3 PathHunter β-arrestin recruitment assays

Assays were carried out using the PathHunter hCB2_bgal CHOK1 β-arrestin recruitment assay kit (DiscoveRx, Fremont, CA, United States) as previously described (Soethoudt et al., 2017). A total of 5,000 cells per well were seeded in 384-well plates (PerkinElmer, Waltham, MA, United States) containing 20 μL cell culture medium and incubated for 16–18 h at 37°C in 5% CO_2_. Cells were stimulated with RG7774 and incubated for 1.5 h at 37°C in 5% CO_2_. As the RG7774 stock solution was 100% DMSO, the final concentration of DMSO per assay point was diluted to 0.1%. PathHunter Detection mixture was used to determine β-galactosidase enzyme activity according to the manufacturer’s protocol. Detection mixture (12 µL per well) was added, and the plate was incubated for 1 h in the dark at RT. Chemiluminescence (indicated as relative light units) was measured on an EnVision multilabel plate reader (PerkinElmer, Waltham, MA, United States).

#### 2.2.4 xCELLigence assay

Background impedance was determined in 96-well RTCA E-plates (Agilent Technologies, Santa Clara, CA, United States) coated with 150 μg/mL fibronectin/H_2_O (Sigma-Aldrich, St. Louis, MO, United States) and containing 50 μL RPMI 1640 assay medium (Fisher Scientific, Schwerte, Germany) 31870) with 10% fetal calf serum (FCS). Jurkat cells (200,000 cells per well) were seeded in 100 μL of growth medium, allowed to settle for 30 min at RT, and cultured overnight at 37°C and 5% CO_2_. Dose-response curves were generated by adding increasing concentrations of RG7774 to seeded Jurkat cells and performing impedance measurements (xCelligence RTCA M, Agilent Technologies, Santa Clara, CA, United States) at baseline and 1 h after treatment. Displacement experiments were performed by incubating seeded Jurkat cells with 10 μM SR144528, a CB2R-selective antagonist (Tocris Bioscience, Bristol, United Kingdom) or 10 μM RO6851228 (a CB2R-selective inverse agonist; in-house synthesis) in assay medium overnight at 37°C and 5% CO_2_. Cells were re-treated with 10 μM SR144528 or RO6851288 for 1 h and stimulated with 233 nM RG7774 in assay medium. Impedance measurements were performed as initially described (Hillger et al., 2015) before the addition of RG7774 and again after 1 h. Normalized cell index measurements after 1 h of compound treatment were used for analysis. Dose-response curve data are presented as the percentage of the maximal normalized cell index of 233 nM RG7774. Displacement data are presented as the percentage of the maximal normalized cell index determined after antagonist/inverse agonist treatment relative to the percentage of the normalized cell index at 109 nM RG7774 treatment.

#### 2.2.5 Phosphorylated extracellular signal-regulated kinase (pERK) assay

ERK phosphorylation in response to RG7774 treatment was measured in Jurkat cells as described in (Saroz et al., 2019) using the AlphaLISA SureFire Ultra p-Erk1/2 assay (PerkinElmer, Waltham, MA, United States). Cells (200,000 per 96 well plate) were seeded in 40 μL RPMI 1640 assay medium (Fisher Scientific, Schwerte, Germany) containing 10% FCS, 2 mM L-Glu, and 25 mM HEPES) and incubated for 1 h at 37°C in 5% CO_2_. Cells were treated with increasing concentrations of RG7774 in 40 μL assay medium. Plates were shaken for 30 s (sec) at 450 rpm, incubated for 2 min at 37°C in 5% CO_2_, and placed on ice. Ice-cold lysis buffer (20 μL 5x) (supplied with the assay kit) was added, plates were shaken (500 rpm) for 10 min at 4°C, and 10 μL of lysate was transferred to a 384-well Optiplate (PerkinElmer, Waltham, MA, United States). The AlphaLISA assay was performed according to the manufacturer’s protocol. Plates were read using Tecan Infinite M1000Pro (excitation 680 nm for 100 ms, integration time 300 ms, and emission 615 nm).

### 2.3 Physicochemical and ADMET studies

The methods used to determine the physicochemical and absorption, distribution, metabolism, elimination, and toxicity (ADMET) properties of RG7774 can be found in Supplementary Methods S2.

### 2.4 Pharmacokinetic (PK) studies in rodents

#### 2.4.1. Animals for PK analysis of RG7774

The plasma PK profile of RG7774 was assessed in adult male C57BL/6J mice and female Wistar rats (Charles River, Cologne, Germany) housed in a controlled environment (temperature, humidity, and 12 h light/dark cycle) with unrestricted access to food and water. The ocular PK profile of RG7774 was assessed in adult female Black Norway rats (Charles River, Mattawan, MI, United States) housed in a controlled environment (21°C–22°C temperature, 40%–60%, humidity, and 12 h light/dark cycle) with unrestricted access to food and water and conducted according to local regulations by EyeCRO (Ann Arbor, MI, United States).

#### 2.4.2. Plasma PK profile of RG7774

In the first study (animal permission #1857), mice received RG7774 as an intravenous (IV) bolus at 1 mg/kg (n = 6) or as an oral (PO) dose at 3 mg/kg (n = 5). In the second study (animal permission #2373), rats received RG7774 as an IV bolus at 10 mg/kg (n = 4) or via gavage at 30 mg/kg (n = 3). Blood samples were collected 0.1, 1, 3, 10, and 24 h post-dose under deep anesthesia with 3%–5% isoflurane in 100% O_2_. Samples were collected sublingually from mice, from the tail vein in rats or via heart puncture (terminal) into anticoagulant-coated polypropylene tubes and placed on ice. Plasma was prepared within 30 min of collection by centrifugation at 3,000 g for 5 min at 4°C and was immediately frozen. RG7774 concentrations in plasma were determined using LC-MS/MS. PK parameters were estimated by non-compartmental analysis of plasma data using Phoenix WinNonlin software (Certara, Princeton, NJ, United States).

#### 2.4.3. Ocular tissue PK of RG7774 in hyperglycemic rats

4 groups of 10 STZ-induced Black Norway rats, received daily oral doses of 1, 3, 10 or 30 mg/kg/day for 30 days. Blood sampling was done following 5 days or 30 days of dosing at 0.25, 1, 3, 6, 10 and 24 h post-dose and vitreous, retina and choroid sampling on D30 at 0.25, 1, 3, 10 and 24 h post last dose in a composite design. Samples were collected from the tail vein or via heart puncture (terminal) and prepared within 30 min of collection by centrifugation at 3,000 g for 5 min at 4°C and were immediately frozen. RG7774 concentrations in plasma and homogenized tissues were determined using LC-MS/MS. PK parameters were estimated by non-compartmental analysis of plasma data using Phoenix WinNonlin software (Certara, Princeton, NJ, United States).

### 2.5 Studies in animal models of retinal diseases

#### 2.5.1 LPS- induced leukocyte adhesion in rodents with endotoxin-induced uveitis (EIU)

Male BALB/c mice aged 8–10 weeks and weighing 21–26 g (Charles River, QC, Canada) were housed in individually ventilated Allentown cages (Allentown Inc., NJ, United States). Maximum density was n = 5 per cage, with the same litter and sex. Male Lewis rats aged 7–10 weeks and weighing 200–300 g (Charles River, QC, Canada) were housed in static cages (Allentown Inc., NJ, United States), n = 2 per cage with the same litter and sex. Both mice and rats were maintained in a controlled environment (21°C–22°C, humidity range 40%–60%, and a 12 h light/dark cycle), with unrestricted access to food and water. All experiments were conducted in accordance with the standards and procedures of the Canadian Council on Animal Care (CCAC, 2024) and were approved by the Dalhousie University Committee on Laboratory Animals. The protocol #13-027 was approved for experimental work in mice. The protocol #14-111 was approved for experimental work in rats. All experiments were performed and are reported in accordance with the ARRIVE guidelines (Kilkenny et al., 2010; McGrath et al., 2010).

##### 2.5.1.1 Creation of EIU rodent models for leukocyte adhesion studies

Mice and rats were anesthetized with 5% isoflurane in 100% O_2_ and intraperitoneal (IP) injection of sodium pentobarbital (65 mg/kg), respectively, before receiving IVT injections of sterile 0.9% saline solution (control) or 125 ng/μL LPS (L8274, Sigma-Aldrich, St. Louis, MO, United States) diluted in 0.9% saline. Injections were administered through the pars plana using a 30-gauge needle and Hamilton syringe (Hamilton Company, Reno, NV, United States) with the assistance of a WILD M37 dissecting microscope (Leitz Canada, Vaughan, ON, Canada). Mice received 2 μL of the LPS solution; rats received 5 μL. To avoid touching the lens or damaging the eye, the tip of the needle was directed toward the posterior pole and only the beveled tip (2–3 mm) was allowed to enter the vitreal cavity. The needle was held in place for 5 s to avoid LPS leakage through the sclerostomy (injection site) and the sclerostomy site was closed using 3M Vetbond Tissue Adhesive (3M Animal Products, St Paul, MN, United States). Animals with intraocular bleeding or swelling post-injection were excluded from EIU experiments. Animal details are summarized in Table 1.

**TABLE 1:** Summary of study arms from *in vivo* efficacy studies with references to methods.

Group/Species		Treatment/RoA		Dose (mg/kg/d)/RoA		n (animals/eyes)	
2.5.1.1. LPS-induced leukocyte adhesion							
Vehicle	Mouse	LPS	ivt	-	-	6	6
RG7774	Mouse	LPS	ivt	0.03	iv	4	4
RG7774	Mouse	LPS	ivt	0.3	iv	5	5
RG7774	Mouse	LPS	ivt	3.0	iv	6	6
Vehicle	Rat	LPS	ivt	-	-	5	5
RG7774	Rat	LPS	ivt	1.0	iv	4	4
2.5.2.2. LPS-induced permeability							
Vehicle	Mouse	PBS	ivt	-	-	6	10
Vehicle	Mouse	LPS	ivt	-	-	6	11
RG7774	Mouse	LPS	ivt	6	gavage	6	11
RG7774	Mouse	LPS	ivt	20	gavage	6	11
2.5.3.2. STZ-induced type 1 diabetes							
Non-diabetic	Rat	-		-	-	15	16
Diabetic vehicle	Rat	STZ	ip	-	-	13	12
RG7774	Rat	STZ	ip	10	gavage	9	13
2.5.4.1. Laser-induced CNV							
Vehicle	Rat	Laser 6x	NA	-		25	23
RG7774	Rat	Laser 6x	NA	0.01	gavage	5	5
RG7774	Rat	Laser 6x	NA	0.03	gavage	5	5
RG7774	Rat	Laser 6x	NA	0.1	gavage	10	10
RG7774	Rat	Laser 6x	NA	0.3	gavage	5	5
RG7774	Rat	Laser 6x	NA	1.0	gavage	10	10
RG7774	Rat	Laser 6x	NA	3.0	gavage	15	13
RG7774	Rat	Laser 6x	NA	10	gavage	15	15

RoA, route of administration, LPS, lipopolysaccharide, STZ, streptocotozin, NA, not applicable, ivt, intravitreal injection, ip, intraperitoneal, CNV, choroidal neovascularization.

##### 2.5.1.2 RG7774 treatment of rodents with EIU

Mixed micelles (vehicle control) [w/w] were prepared using 9.4% glycocholic acid, 15.7% lecithin, and 3.8% NaOH (pH 6.5). RG7774 was added to freshly prepared mixed micelles, stirred for up to 2 h at RT until the suspension cleared, and filtered using a 0.2-micron filter. Mice received 0.1 mL IV injections of vehicle control or RG7774 (0.03 mg/kg, 0.3 mg/kg or 3 mg/kg). Vehicle control and RG7774 at doses of 0.3 and 3 mg/kg were administered via tail vein injection while mice were awake but restrained. RG7774 0.03 mg/kg was administered via penis vein injection while mice were anesthetized under isoflurane. Rats received 0.3 mL injections of vehicle or RG7774 1.0 mg/kg into the femoral vein. All injections were administered 15 min after the IVT injection of LPS.

##### 2.5.1.3 Intravital video microscopy (IVM) in rodents with EIU

IVM was used for *in vivo* investigations of LPS-induced leukocyte recruitment as previously described (Toguri et al., 2014). Mice and rats with EIU were weighed prior to anesthetizing with IP sodium pentobarbital (65 mg/kg; Ceva Sante Animale, Montreal, QC, Canada). The depth of anesthesia throughout the procedure was assessed by the absence of a toe-pinch reflex. If needed, “top-up” pentobarbital was administered up to a total dose of 54 mg/kg. Anesthetized animals were transferred to a heating pad and Tear-Gel^®^ was regularly applied to the eyes to prevent drying of the corneal surface. Leukocytes were stained *in vivo* using IV injections of fluorochrome dyes comprising rhodamine 6G (1.5 mL/kg, 0.75 mg/kg body weight) (Sigma-Aldrich, St. Louis, MO, United States) and fluorescein isothiocyanate (FITC-conjugated to albumin) (1 mL/kg, 50 mg/kg body weight) (Sigma-Aldrich, St. Louis, MO, United States). The intravital fluorescence video microscope (Olympus OV 100 Small Imaging System, Richmond Hill, ON, Canada) was focused on the iridial microcirculation to image leukocyte-endothelial interactions in iridial venules. Venules were identified by the direction of blood flow moving away from the pupil. Throughout IVM, the animal’s head was held stationary in a rotational head holder and a cover slip was placed over the left eye to facilitate imaging and prevent desiccation of the cornea. For imaging, the iris was divided into 4 equal quadrants by drawing 2 artificial lines: 1 lengthwise and the other widthwise. IVM was carried out at each of these quadrants. In each video, leukocyte recruitment was observed and recorded for 30 s. Evaluation of the videos was carried out offline. All animals were sacrificed after IVM by spinal cord dislocation and eyes were enucleated for further tissue preparations. Results were analyzed using Prism 5 software (GraphPad, La Jolla, CA, United States). All data are expressed as mean ± standard error of the mean (SEM). The normality of distribution was tested using the Shapiro-Wilk test and showed normal distribution of data. IVM data from mice were tested for significance (*p* < 0.05) using one-way analysis of variance (ANOVA) with a Bonferroni *post hoc* test, comparing all experimental groups to the vehicle-treated group. Every possible comparison between experimental groups was considered. IVM data from rats were analyzed using an unpaired *t*-test between vehicle and the single treatment group.

#### 2.5.2 Vascular permeability studies in mice with EIU

Twenty-four female C57Bl6/J mice, 10 weeks old and weighing 18–20 g (Charles River, Sulzfeld, Germany) were housed in individually ventilated GM500 cages (Tecniplast, Pontremoli, Italy) with a maximum density of n = 4 mice with the same litter and sex. The room was kept at 20°C–24°C with 50%–60% humidity and a 12/12 h light-dark cycle. Animals were held on a standard mouse diet with *ad libitum* access to food and water.

##### 2.5.2.1 EIU induction in mice for vascular permeability studies

Mice were anesthetized with isoflurane 3% in 100% O_2_, a drop of 1% Novesin 0.4% (Omnivision, Pucheim, Germany) and a drop of Tropicamide 1% w/v (Bausch + Lomb Incorporated, Kingston, United Kingdom). Eyes were subsequently cleaned with povidone iodine 5% Ophthalmic Prep Solution (Alcon, Geneva, Switzerland) and were immediately pre-punctured with a 30-gauge needle. Both eyes were injected with a single 1 µL IVT injection of 1 ng LPS (Sigma-Aldrich, St. Louis, MO, United States) or a single injection of Dulbecco’s phosphate buffered saline (PBS) (Fisher Scientific, Schwerte, Germany) (vehicle control) using a Nanofil syringe connected to a 34-gauge needle.

##### 2.5.2.2 RG7774 treatment of mice with EIU

Vehicle solution [w/w] was prepared using 0.1% hydroxyethyl cellulose, 1.0% polysorbate 80, 0.18% methylparaben, and 0.02% propylparaben in 10 mM citrate (pH 6.0). RG7774 (0.6 and 2.0 mg/mL) was added to freshly prepared vehicle, stirred on a magnetic stirrer for at least 15 min, and a volume of 5 mL/kg was administered directly to the stomach of each EIU mouse using a 1 mL syringe with an oral gavage needle. Gavage was performed twice daily (6 mg/kg/day BID and 20 mg/kg/day BID) on the evening and morning prior to LPS injection, on the evening after LPS injection, and on the morning before fluorophotometry. Animal details are summarized in Table 1.

##### 2.5.2.3 Retinal vascular permeability assessments in mice with EIU

Mice were anesthetized 24 h after receiving LPS or vehicle control using subcutaneous (SC) injections of fentanyl 0.05 mg/kg, medetomidine 0.5 mg/kg, and midazolam 5 mg/kg. Subcutaneous fluorescein solution (1%, 10 mg/mL; Sigma-Aldrich, St. Louis, MO, United States) was administered at a dose of 5 mL/kg body weight in a total volume of approximately 100 µL/mouse. Fundus infrared and fundus fluorescein angiography (FFA) images of both eyes were recorded 30 min later using optical coherence tomography (OCT)/FFA (Spectralis HRA-OCT, Heidelberg Engineering, Heidelberg, Germany) with a 55° lens focused on the optic nerve with the optic disc in the center of the image to obtain sharp images of the fundus. Fluorophotometry was conducted to assess retinal vascular permeability by placing fluorescein-injected mice on a temperature-controlled (37°C) fluorophotometer stage (FM-2 Fluorotron™ Master, OcuMetrics, Mountain View, CA, United States), and adjusting the stage to align the eyes with the optic device. Both eyes were scanned 1 h after fluorescein injection (450–490 nm excitation; 520–600 nm emission detection). To correct for fluorescein in the circulation, a 25 µL blood sample was taken after fluorophotometry, transferred to a microcuvette containing EDTA (Sarstedt AG, Nümbrecht, Germany) and plasma was obtained by centrifugation at 10,000 g for 10 min. The supernatant (10 µL) was diluted in 990 µL PBS in a microcuvette (PS Micro Photometer Cuvette 2 mL, 10 mm × 10 mm × 45 mm; LP Italiana, Milan, Italy) and samples were analyzed in duplicate using the FM-2 Fluorotron (450–490 nm excitation; 520–600 nm emission detection). An average of 2 separate measurements was used to normalize ocular measurements against the amount of fluorescein circulating in the blood. The amounts of fluorescein in the retina, vitreous, and anterior chamber eye compartments were expressed in ng/ml as a ratio of plasma fluorescein (Colé et al., 2023).

To quantify fluorotron measurements, raw files were generated, exported as txt. files, and plotted using Microsoft Excel. The average area under the curve (AUC) was calculated for 5 steps in each of the regions of the scans corresponding to retina, vitreous, and anterior chamber peaks. In addition, the amount of fluorescein in each animal’s plasma was calculated by averaging the maximum peak values of duplicate measurements for each plasma sample. Fluorescein levels in each eye compartment were normalized to the amount of fluorescein circulating in the blood of each mouse to account for potential administration differences. All data are expressed as mean ± SEM ng/mL ratios of ocular compartment:plasma fluorescein. Data analysis was carried out using Microsoft Excel (Microsoft Corporation, Redmond, WA) and Prism 5 software (GraphPad, La Jolla, CA, United States). Normality of data were confirmed with Shapiro-Wilk *post hoc* test. ANOVA (alpha 0.05) followed by Newman–Keul’s multiple comparisons test were used for each analysis. Every possible comparison between study groups was considered. Outliers were removed from the dataset prior to statistical analysis using Chauvenet’s criteria (±1.74 standard deviations [SD]). All personnel involved in data acquisition were blinded to the treatment groups until the raw data were fully processed. Treatment groups were not identified until all analyses were complete.

#### 2.5.3 Studies in rats with STZ-induced type 1 diabetes

Twenty-six male Long Evans rats (outbred) (Charles River Laboratories, Sulzfeld, Germany) aged 7 weeks and weighing 250–270 g at the beginning of the study were housed in individually ventilated cages with a maximum density of n = 2 rats per cage with the same sex and monitored daily. Bedding for diabetic animals was changed daily. The room was kept at 20°C–24°C with 50%–60% humidity and a 12/12 h light-dark cycle. Rats received a standard diet and water ad libidum, in addition to soaked food or soft diet. For animal welfare and for dosing purposes, animals were weighed every other day. Rats were sacrificed if weight loss was greater than 20% of the original weight, or more than 20% of body gain within 1 week.

##### 2.5.3.1 Diabetes induction

Animals were fasted overnight and injected the following morning with IP 60 mg/kg (60 mg/mL at 1 mL/kg) STZ as reported previously (Kim et al., 2007; Furman, 2015) in 0.05 M sodium citrate buffer (pH 4.5). Blood glucose levels were checked again in fasted animals in the morning (from approximately 8 a.m.) 5 days after injection and animals were considered diabetic if levels exceeded 13.8 mmol/L. Glucose levels in nondiabetic animals were checked again 2 days later but were not reinjected with STZ. Weekly glucose checks from non-fasted animals were conducted by tail vein puncture using Alphatrak strips and glucometer (Zoetis, Parsippany, NJ, United States). Nondiabetic rats that had received vehicle 0.05 M sodium citrate buffer (pH 4.5) injections instead of STZ were used as controls.

##### 2.5.3.2 RG7774 treatment of diabetic rats

RG7774 (10 mg/kg) was stirred for ≥ 15 min with freshly prepared vehicle solution [w/w] (1.25% hydroxypropyl methylcellulose, 0.1% docusate sodium, 0.18% methylparaben, 0.02% propylparaben, 0.21% citric acid monohydrate pH 6, and purified water ad 100%). Rats received daily 5 mL/kg treatments of either vehicle control or 10 mg/kg RG7774 by oral gavage directly to the stomach using a 1 mL syringe with an oral gavage needle for 5 weeks. Animal details are summarized in Table 1.

##### 2.5.3.3 Fluorophotometry in diabetic rats

Fluorophotometry was performed 6 weeks after STZ injection (week 5 of RG7774 treatment). Rats were anesthetized using 3%–5% isoflurane with 100% O_2_, and fluorescein (Martindale Pharma, Bucks, United Kingdom) was administered via IV injection into the tail vein at a dose of 50 mg/kg in a total volume of 1 mL/kg. Injected rats were placed on a temperature-controlled (37°C) stage in front of the fluorophotometer (Fluorotron™ Master, OcuMetrics, Mountain View, CA, United States) and animals were positioned so the eye was parallel to the optic device. Measurement sequences were started 1 h after fluorescein injections and both eyes were scanned (450–490 nm excitation; 520–600 nm emission detection).

To quantify fluorotron measurements, raw data were generated, exported as .txt files, and plotted using Microsoft Excel. The average AUC was calculated for 5 steps in the region of the scans corresponding to the vitreous peak. A 25 µL blood sample was taken from each animal after fluorometry and plasma was prepared by centrifugation at 10,000 g for 10 min. Fluorescein levels in each eye compartment were normalized against fluorescein levels in the blood. All data are expressed as mean ± SEM ng/mL ratios of vitreous:plasma fluorescein. Fluorophotometry data were analyzed using 2-way ANOVA followed by Newman–Keuls multiple comparisons test. Every possible comparison between the study groups was considered. Outliers were removed from the dataset before and after combining the 2 datasets using Chauvenet’s criteria (+/− 1.74 SD from the mean) prior to statistical analyses. All personnel involved in data acquisition were blinded to treatment groups until raw data were fully processed. Treatment groups were not identified until all analyses were complete.

#### 2.5.4 Studies in rats with laser-induced CNV

Male Brown Norway rats (Charles River, Sulzfeld, Germany) aged 9 weeks and weighing 200 g on arrival (230–250 kg after 1 week) were housed in individually ventilated GR1800 cages (Tecniplast, Pontremoli, Italy) with a maximum density of n = 5 rats per cage with the same litter and sex. The room was kept at 20°C–24°C with 50%–60% humidity and a 12/12 h light-dark cycle with *ad libitum* access to standard diet and water.

##### 2.5.4.1 RG7774 treatment of rats

RG7774 was formulated (w/w) in 0.1% hydroxyethyl cellulose, 1% polysorbate 80, 0.18% methylparaben, 0.02% propylparaben, and 0.21% citric acid monohydrate, adjusted to pH 6 using NaOH. Three studies with 20 rats each were performed. Rats received oral gavage at the same time each day (8 a.m.) with doses of 10, 3, and 1 mg/kg (study 1), 1, 0.3, and 0.1 mg/kg (study 2), or 0.1, 0.03, and 0.01 mg/kg (study 3). The wellbeing of the animals was monitored daily, and free access was granted to food and water. Treatment started 1 day prior laser injury and was continued daily until day 7. Body weight was continuously monitored, and oral doses of RG7774 were adjusted accordingly. Animal details are summarized in Table 1.

##### 2.5.4.2 Laser-induction of CNV

Rats received their daily oral dose of RG7774 in the morning and were anesthetized at least 30 min later using fentanyl 0.005 mg/kg, medetomidine 0.15 mg/kg, and midazolam 2 mg/kg. Eyes were dilated using 1% tropicamid, covered and kept humid with Viscotears^©^, a protective gel (Bausch + Lomb, Zug, Switzerland) to avoid corneal drying. Laser injury was performed in both eyes (6 shots per eye) using an image guide laser photocoagulation system (600 mW, 100 ms; Micron IV, Phoenix Research Labs, Pleasanton, CA, United States). Laser lesions were 2–2.5-fold greater than the size of the optic nerve disc from the center. Rats were woken up after laser injury and at least 40 min after the onset of anesthesia using naloxone 0.12 mg/kg, atipamezol 0.75 mg/kg, and flumazenil 0.2 mg/kg.

##### 2.5.4.3 Fluorescein angiography in eyes with CNV

Rats were anesthetized 1 week after laser injury using fentanyl 0.005 mg/kg, medetomidine 0.15 mg/kg, and midazolam 2 mg/kg. Eyes were dilated with tropicamide 1% and protected against drying as previously described. Rats received IV tail vein injections of fluorescein 40 mg/kg (20 mg/mL solution) in 0.9% NaCl (approximately 0.5 mL/rat) and were placed in front of the Heidelberg Spectralis scanner (HRA-OCT, Heidelberg Spectralis, Heidelberg Engineering, Heidelberg, Germany) on an adjustable table. Existing and newly formed vessels appeared white in the FA mode. Laser lesion locations were visualized using an infrared mode, focusing on the fundus. Retinal vessels and neovascularization were visualized by switching to fluorescence mode.

Images taken by the Heidelberg Spectralis HRA system were manually analyzed for the degree of CNV within the lesion (fluorescent) area and the affected area (in mm^2^) was calculated using the Heidelberg Eye Explorer imaging program (Heidelberg Spectralis HRA, Heidelberg Engineering, Heidelberg, Germany). Images taken before and after analysis (with the respective overlay of measured areas) were exported from the local imaging program in JPG format to the respective experimental folder. Lesion areas (in mm^2^) per eye were transferred to Microsoft Excel and calculated as single shots (mean 6 lesions per eye, only sharp images were used for analysis) and mean per group. Data were transferred to Prism 5 (GraphPad, La Jolla, CA, United States) for statistical analysis.

##### 2.5.4.4 Histological analyses of eyes with CNV

Right eyes were transferred to a petri dish containing 10% neutral-buffered formalin (NBF) and fixed for 5 min at RT. Eyes were prepared in PBS^−/–^ by removing the anterior segments and carefully separating the retinas from the posterior segments (retinal pigment epithelium/choroid and sclera). Dissected retinas were cut into petals, transferred to 2 × 24 well plates containing fresh 10% NBF solution, and fixed for 2 h at RT. NBF was replaced with Tris-buffered saline (TBS) and retinas were washed 3 times before incubating with CASHBLOCK (0.1% sodium azide, 0.1% cold water fish skin gelatin, 1% BSA, 2% donkey serum, 0.05% TBS with Tween-20, 0.5% Triton X-100) over night at 4°C with gentle agitation. Retinas were incubated with a first antibody cocktail consisting of biotin-conjugated isolectin B4 1:100 (1 mg/mL) (L2140, Sigma-Aldrich, St. Louis, MO, United States) and rabbit pAB anti-Iba1 1:400 (019-19741, FUJIFILM Wako Chemicals Europe, Neuss, Germany) for 4 h at RT on a shaker and then with a second cocktail consisting of DyLight 488 Streptavidin 1:100 (1 mg/mL, SA-5488, Vector Laboratories, Newark, CA, United States) and Alexa Fluor 555 1:200 (0.5 mg/mL, A31572, Fisher Scientific, Schwerte, Germany) for 1.5 h at RT on a shaker. Retinas were washed with TBS containing 0.05% Tween 20% and 0.5% Triton X-100 and flat mounted on SuperFrost plus adhesion slides (ThermoFisher Scientific, Rochford, IL, United States) using fluorescent mounting medium (Agilent Dako, Santa Clara, CA, United States). Confocal images of flat mounted retinal tissue were acquired using a Zeiss Confocal LSM710 using a Plan-Apochromat 20x/0.8 M27 objective at 488 nm (IB4) and 561 nm (Iba1). Up to 39 consecutive confocal image frames taken in Z-direction (1,024 × 1,024 pixel, i.e., 0.415 µm × 0.415 µm per frame, distance in Z-direction between frames was 1.5 µm) were acquired as CZI-files that spanned the retina from nerve fiber.

##### 2.5.4.5 Semi-automated image analysis and quantitation of microglia volume on laser lesions

Microglia migration towards laser-injured retinal regions was quantified from 3D RGB-images (CZI-files) of the fluorescently labeled retinal samples, a workflow and image analytics tool was applied that allowed a semi-automated volumetric quantitation of Iba1-positive pixel per image frame integrated over the Z-dimension. 3D CZI-files were exported by ZEN 2 lite software (blue edition, Carl Zeiss Microscopy GmbH, Oberkochen, Germany) into separated frames in TIFF format. Subsequently, TIFF images were analyzed using IDL (Interactive Data Language, Version 6.4) in combination with Excel 2010 to compute RGB-channel-dependent threshold images (pdf-files) with a histogram between 1–255 A.U. pixel intensity. These pdf -files were used to determine a custom-defined threshold. The sum of pixels displaying the determined threshold intensity or higher was calculated and finally integrated over the Z-dimension of the 3D image.

## 3 Results

### 3.1 RG7774-CB2R modeling studies

**FIGURE 2:**
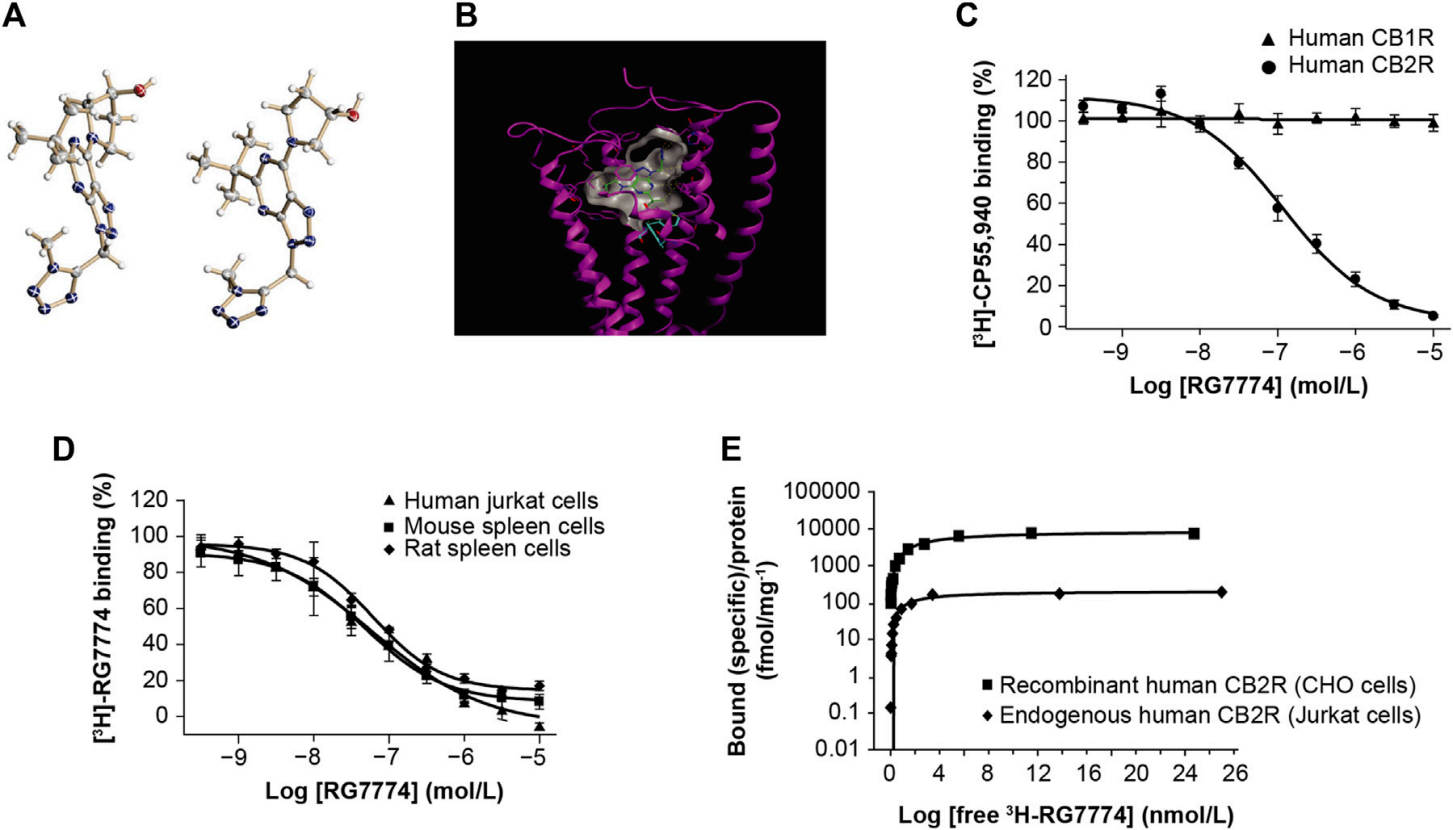
Molecular structure of **(A)** RG7774 determined by X-ray crystal structure analysis and **(B)** RG7774 docked into the X-ray crystal structure of cannabinoid 2 receptor (CB2R) (PDB: 6KPF). **(C)** RG7774 binding affinity for recombinant human CB2R versus cannabinoid 1 receptor (CB1R) expressed in Chinese hamster ovary (CHO) cells labeled with the CB1R/CB2R-selective agonist, [^3^H]-CP55940. **(D)** RG7774 binding affinity for endogenous CB2R expressed in human, mouse and rat cell lines labeled with the CB1R/CB2R-selective agonist, [^3^H]-RO6753361; **(E)**. RG7774 maximal binding affinity (B_max_) in cells expressing recombinant or endogenous human CB2R. In **(A)**, thermal ellipsoids (drawn at the 50% probability level) for non-hydrogen atoms (heteroatoms N, O, are colored in blue, red, respectively). Two molecules are contained in the asymmetric unit. The absolute configuration of the tertiary carbon atom was determined as (S). In **(B)**, dashed lines indicate favorable interactions between RG7774 and CB2R, and cyan sticks indicate the single ‘‘toggle switch,’’ Trp6.48 (W258) and Phe3.36 (F117).

The molecular structure of RG7774 (Figure 2A) was docked into the previously reported X-ray crystal structure of CB2R in its active state (PDB: 6KPF) (Hua et al., 2020) (Figure 2B). The docking pose and interaction analysis suggest the ligand fits well into the agonist-binding cavity and makes a large number of favorable contacts with CB2R. These include van der Waals contacts between the *tert*-butyl moiety of RG7774 and CB2R residues F117 (Phe3.36), Val261, Cys288, and the single “toggle switch” residue, W258 (Trp6.48), which triggers receptor activation.

### 3.2 *In vitro* binding and selectivity

Competition binding assays using [^3^H]-CP55940 show that RG7774 binds recombinant human CB2R in CHO cells with a dissociation constant (Ki) of 51.3 ± 16.2 nM, with no binding to recombinant human CB1R (Figure 2C). This indicates a > 195-fold binding selectivity for recombinant human CB2R over CB1R. Further studies using [^3^H]-RO6753361 show that the binding affinity of RG7774 for endogenous CB2R in human Jurkat cells (Ki: 31.9 ± 13.4 nM) was almost identical to the affinities observed for endogenous CB2R in rat (Ki: 33.3 ± 8.0 nM) and mouse (Ki: 39.7 ± 13.5 nM) spleen tissues (Figure 2D). Saturation binding studies showed a similar maximum binding capacity (Bmax) for endogenous CB2R in all 3 cell types (0.20, 0.26, and 0.15 pmol/mg protein, respectively), and an approximate 40-fold higher Bmax in CHO cells expressing recombinant human CB2R (8,860 pmol/mg protein). Results in Jurkat and CHO cells are shown in Figure 2E.

Competition binding assays using [^3^H]-CP55940 and mouse brain membranes expressing endogenous CB1R found that RG7774 up to a concentration of 10 µM inhibited CB1R-specific binding only by 3.8% ± 12.5% compared to 112.2% ± 5.6% for the CB1R-selective inverse agonist, rimonabant (Supplementary Methods S3; Supplementary Table S2). The high level of RG7774 selectivity for CB2R was further demonstrated by *in vitro* binding assays for 78 non-endocannabinoid receptors, ion channels, and transporters (CEREP, Celle l’Evescault, France; http://www.cerep.fr) (Supplementary Table S3), and 11 endocannabinoid enzymes, including human 5-, 12-, or 15-lipoxygenase (LOX), monoacylglycerol lipase (MAGL), diacylglycerol lipase (DAGL), endocannabinoid membrane transporter (EMT) or fatty acid amide hydrolase (FAAH), and mouse MAGL, DAGL, or FAAH (Supplementary Methods S4–S9; Supplementary Table S2).

### 3.3 *In Vitro* functional CB2R activation and selectivity

**FIGURE 3:**
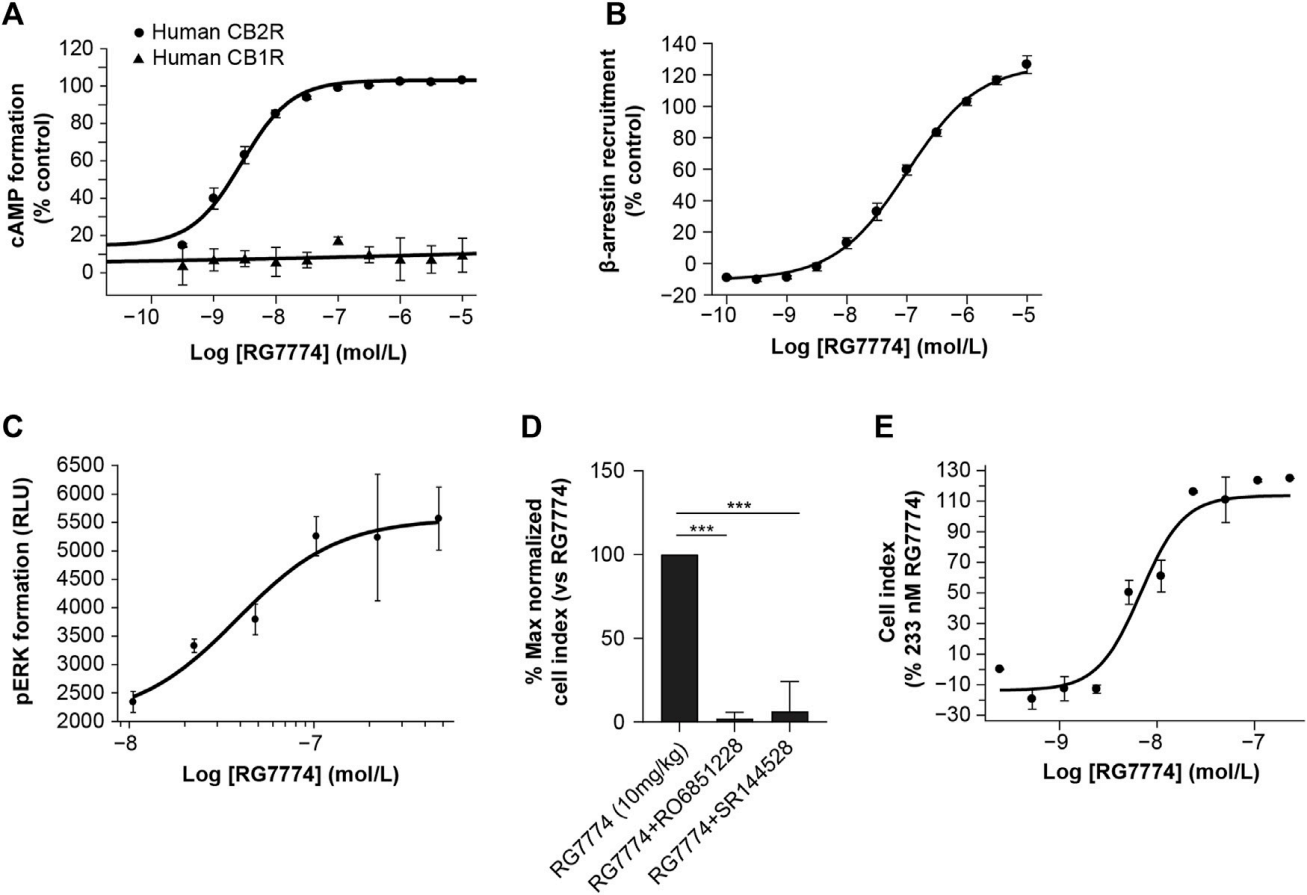
Dose-dependent effects of RG7774 on **(A)** forskolin-induced cyclic adenosine monophosphate (cAMP) levels in Chinese hamster ovary (CHO) cells expressing recombinant human cannabinoid 1 receptor (CB1R) or cannabinoid 2 receptor (CB2R) and **(B)** CB2R-dependent β-arrestin recruitment levels in CHO cells expressing recombinant human CB2R. **(C)** Dose-dependent effects of RG7774 on phosphorylated extracellular signal-regulated kinase (pERK) levels 2 min after incubating RG7774 with Jurkat cells expressing endogenous human CB2R. **(D)** Effects of RG7774 at 109 nM on the percentage maximal normalized cell index measured using the xCELLigence system (54) in Jurkat cells pretreated with 1uM of RO6851228 (inverse CB2R agonist) or SR144528 (CB2R selective antagonist), and **(D)** cell index dose-response curve for RG7774 relative to the maximal cell index of RG7774 at 233 nM, determined using the xCELLigence system (54). For **(D,E)**, results are expressed as mean ± standard error of the mean (SEM) of 3 independent experiments with 4 technical replicates. ***p < 0.001 (1-way ANOVA, followed by Newman–Keuls *post hoc* analysis). RLU: relative light units.

RG7774-mediated inhibition of forskolin-induced cAMP in recombinant CHO cells showed that, compared to the maximum efficacy of the reference agonist (CP55940), RG7774 is a potent, full agonist for recombinant human and mouse CB2R (EC_50_: 2.81 ± 0.28 nM and 2.60 ± 0.14 nM, respectively), with no effect on human CB1R (Figure 3A). Results suggest a >3,600-fold greater functional affinity for human CB2R over CB1R. Further studies in mouse N18TG2 cells expressing endogenous CB1R found that RG7774 at a concentration of 10 µM reduced forskolin-stimulated cAMP levels only by 16.6% ± 23.5% when normalized to the effect of the reference agonist, CP55940 (0.1 µM) (Supplementary Methods S10; Supplementary Table S2). The potency of RG7774 in CB2R-dependent β-arrestin recruitment assays in CHO cells expressing human CBR2 was lower than that observed in cAMP assays (EC_50_: 99.69 ± 5.72 nM) (Figure 3B).

RG7774-mediated CB2R activation did not inhibit forskolin-stimulated intracellular cAMP levels in human Jurkat cells expressing endogenous CB2R (data not shown). However, incubation of Jurkat cells with RG7774 led to a rapid and transient production of perk that peaked after 2 min (data not shown). At that time point, RG7774-mediated increases in pERK were dose-dependent (Figure 3C) with an EC_50_ of 38.5 ± 15.0 nM. Label-free and real-time monitoring of cell morphology using the xCELLigence system (Hillger et al., 2015) showed that the treatment of Jurkat cells with RG7774 significantly increased the maximal normalized cell index, an effect that was fully inhibited following co-treatment with the CB2R-selective antagonist, SR144528, and the inverse agonist, RO6851228 (Porter et al., 2019) (Figure 3D). Dose-response curves for RG7774 relative to the maximal cell index of RG7774 at 233 nM provide an EC_50_ of 10.8 ± 4.2 nM (Figure 3E).

### 3.4 Physicochemical and ADMET properties

The physicochemical and ADMET properties of RG7774 are summarized in Table 2.

**TABLE 2:** Physicochemical and ADMET profile for RG7774.

*M*_*r*_ [Da]	358.408
PSA [Å^2^]	115.9
log*D*_7.4_	2.2
LLE	7.1
LYSA [µg/mL]	285
THESA/FaSSIF/FeSSIF [µg/mL]	901/1177/2872
pKa	2.63 (basic)
PAMPA *P*_*eff*_ [10^−6^ cm s^−1^]	9.7 (23/18/59)^a^
Chemical stability in aqueous media at pH 1–10	stable
Microsomal CL_int_ [µL min^−1^ (mg protein)^−1^] (human/mouse/rat)	21.7/15.0/25.0
Hepatocyte CL [µL min^−1^ (10^−6^ cells)^−1^] (human/mouse/rat)	1.7/49.3/37.9
PPB (fu) [%] (human/mouse/rat)	17.8/4.5/15.8
P-gp efflux ratio (human/mouse)	2.5/16.2
GSH (human liver microsomes) adducts	none detected
CYP inhibition [µM] (3A4/2C19/2C8/2C9/2D6/1A2)	>50/>50/>50/29/>50/>50
hERG IC_50_ [µM]	>20

^a^Acceptor compartment/membrane/donor compartment [%]. Å: Angström; ADMET: absorption, distribution, metabolism, elimination and toxicity; CL: clearance; CL_int_: intrinsic clearance; CYP: cytochrome P-450; Da: daltons; FaSSIF: fasted simulated gastrointestinal fluid; FeSSIF: fed simulated gastrointestinal fluid; fu: fraction unbound; GSH: glutathione-stimulating hormone; hERG: human Ether-à-go-go-related gene; IC_50_: half maximal inhibitory concentration; LLE: ligand-lipophilicity efficiency; logD: octanol/water distribution coefficient; LYSA: lyophilization solubility assay; *M*_*r*_: molecular weight; PAMPA: parallel artificial membrane permeability assay; P-gp: permeability glycoprotein; pKa: acid-base dissociated constant; PSA: polar surface area; THESA: thermodynamic solubility assay.

### 3.5 Systemic and ocular PK in healthy rodents

**FIGURE 4:**
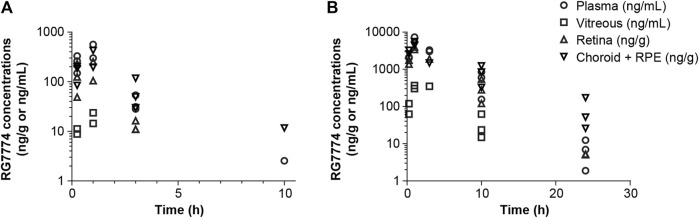
Concentrations of RG7774 in plasma and ocular tissues 26 days after daily oral administration of **(A)** RG7774 3 mg/kg/day and **(B)** 30 mg/kg/day in rats. RPE: retinal pigment epithelium.

The PK parameters of RG7774 in healthy mice and rats after IV and oral administration are shown in Table 3. Following 26 days of oral RG7774 administrations, concentrations in retina and choroid/retinal pigment epithelium tissues of rats were in the same range as the total plasma concentration, while exposure in the vitreous humor was closer to that found in free plasma (Figure 4). The PK studies provided exposure data that informed dose selection for subsequent rodent models. Specifically, doses of 1 mg/kg (intravenous) and 3 mg/kg (oral gavage) in mice or 10 mg/kg (intravenous) and 30 mg/kg/d (oral gavage) in rats achieved adequate plasma and ocular concentrations to encompass the CB2R Ki of RG7774.

**TABLE 3:** PK parameters of RG7774 following a single IV or PO dose in healthy mice and rats.

Route	IV		PO	
	Mice (n = 6*)	Rats (n = 4)	Mice (n = 5*)	Rats (n = 3)
Dose (mg kg^–1^)	1	10	3	30
Cmax/dose [ng ml^–1^ (mg kg^–1^) ^−1^]	-	-	487	466 (13)
Tmax (h)	-	-	0.25	1.25 (35)
AUCinf/dose [ng h^–1^ ml^–1^ (mg kg^−1^) ^−1^]	1,230	6,000 (16)	490	4,670 (9.4)
T1/2 (h)	9.04	2.54 (11)	2.10	3.18 (14)
Vss (L kg^–1^)	3.70	0.442 (15)	-	-
Clearance (mL min^–1^ kg^–1^)	13.2	2.84 (17)	-	-
Bioavailability (%)	-	-	42.2	81.4 (7.9)

Composite data. Values are presented as mean (% coefficient of variance) if n ⩾ 3. AUC_inf_: area under the curve from the time of dosing to infinity; C_max_: maximum concentration; IV: intravenous; PO: oral; T_1/2_: half-life; T_max_: time to maximum concentration; Vss: apparent volume of distribution at steady state.

### 3.6 Effects of RG7774 on LPS-induced vascular permeability *in vivo*

**FIGURE 5:**
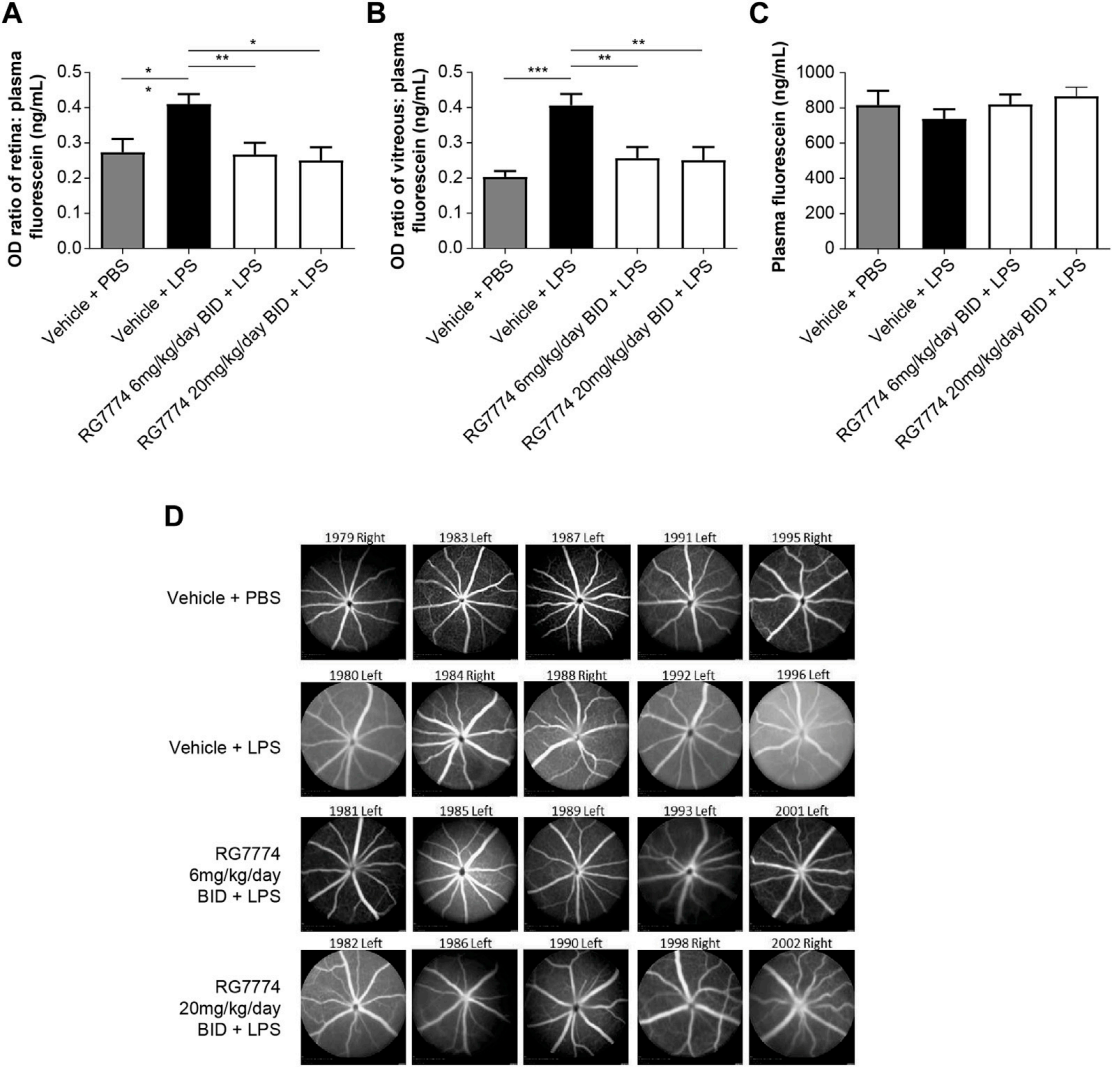
Ratios of **(A)** retina:plasma (n = 11 eyes for all treatment groups) and **(B)** vitreous:plasma, (n = 10 eyes for the vehicle plus phosphate-buffered saline (PBS) group, n = 11 eyes for all other treatment groups), and **(C)** plasma levels of fluorescein (n = 6 mice) in mice treated with and without intravitreal (IVT) lipopolysaccharide (LPS) and/or twice daily (BID) oral RG7774 (6 mg/kg/day or 20 mg/kg/day). **(D)** Representative fundus fluorescein angiography (FFA) images (Heidelberg Spectralis) of study eyes from mice treated with and without LPS and/or twice daily oral RG7774 (6 mg/kg/day or 20 mg/kg/day). In **(D)**, mouse identification numbers and left/right eye labels are shown above each image. *p < 0.05; **p < 0.01; ***p < 0.001 based on 1-way analysis of variance (ANOVA), followed by Newman–Keuls *post hoc* analysis.

Fluorophotometry studies show that IVT injection of LPS in mouse eyes resulted in a significant 50% increase in vascular permeability in the retina (Figure 5A) and a 100% increase in the vitreous (Figure 5B) compared to vehicle control (*p* < 0.05 and *p* < 0.001, respectively). Twice daily oral treatment with RG7774 at doses of 6 mg/kg/day and 20 mg/kg/day significantly reduced LPS-induced vascular leakage in the retina by 102% (*p* < 0.01) and 106% (*p* < 0.05), respectively, versus vehicle control (Figure 5A) and by 74% and 76%, respectively, in the vitreous (Figure 5B). No significant differences were observed between RG7774 doses. Plasma fluorescein levels obtained 1 h after SC injection were similar across treatment groups (Figure 5C), indicating that RG7774 had no influence on the systemic plasma clearance of fluorescein. The effects of RG7774 on vascular leakage observed in quantitative fluorophotometry studies are supported by representative FFA images (Figure 5D).

### 3.7 Effects of RG7774 on LPS-induced leukocyte adhesion *in vivo*

**FIGURE 6:**
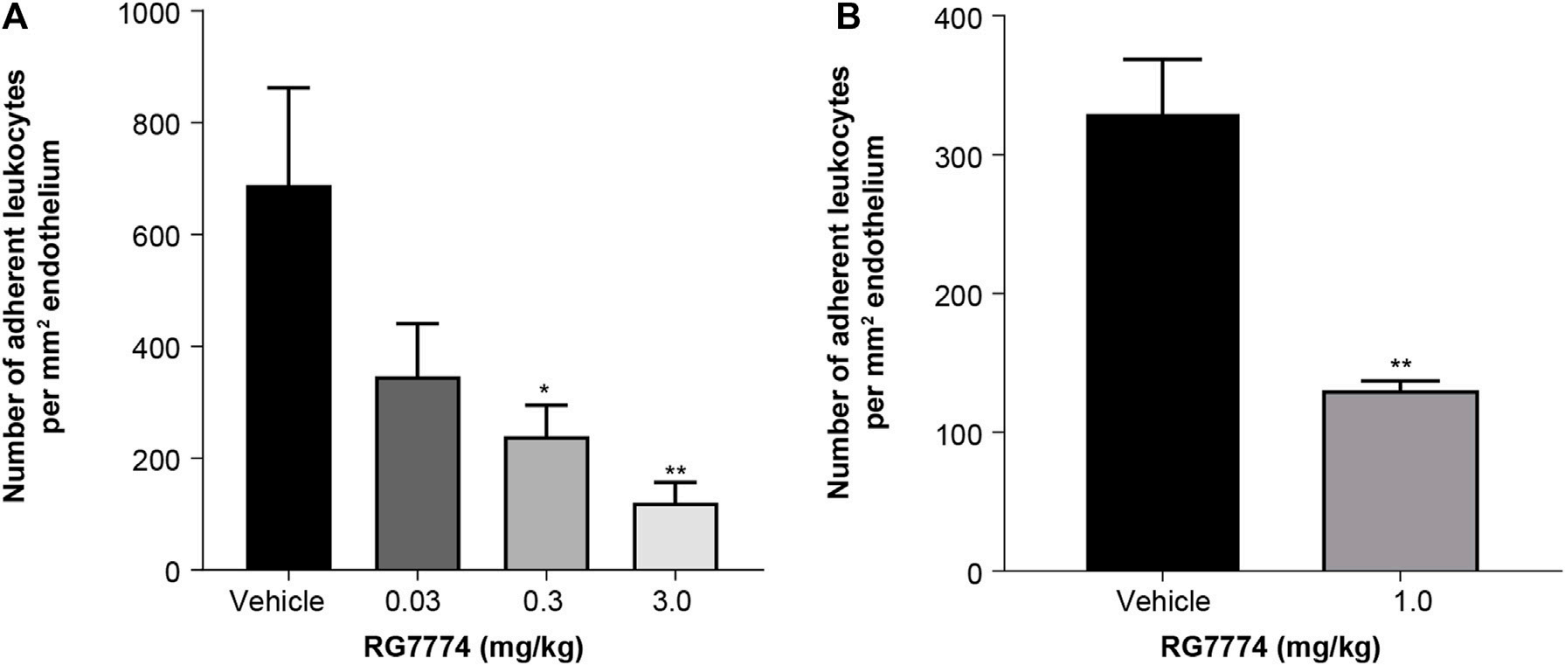
Effects of a single intravenous (IV) injection of vehicle control (n = 6 eyes and n = 5 eyes, respectively) or **(A)** RG7774 0.003 mg/kg (n = 4 eyes), 0.3 mg/kg (n = 5 eyes) or 3.0 mg/kg in mice (n = 6 eyes) with lipopolysaccharide (LPS)-induced endotoxin-induced uveitis (EIU) and **(B)** RG7774 1.0 mg/kg (n = 4 eyes) in rats with LPS-induced EIU. * <0.05; **p < 0.01; ***p < 0.001 based on 1-way ANOVA, followed by Newman–Keuls *post hoc* analysis.

IVM was used to visualize and quantify adherent leukocytes in the mouse iridial microcirculation 6 h after IVT injection of LPS. A single IV administration of RG7774 0.03 mg/kg, 0.3 mg/kg or 3 mg/kg dose-dependently decreased leukocyte adhesion (21%; *p* = not significant, 65%; *p* < 0.05 and 82%; *p* < 0.01, respectively) compared to vehicle control (Figure 6A). Further studies in a rat model of EIU showed a significant (60%; *p* > 0.001) decrease in leukocyte adhesion 6 h after LPS injection in rats treated with RG7774 at the dose of 1.0 mg/kg compared to vehicle control (Figure 6B).

### 3.8 Effects of RG7774 on retinal vascular permeability in rats with STZ-induced diabetes

**FIGURE 7:**
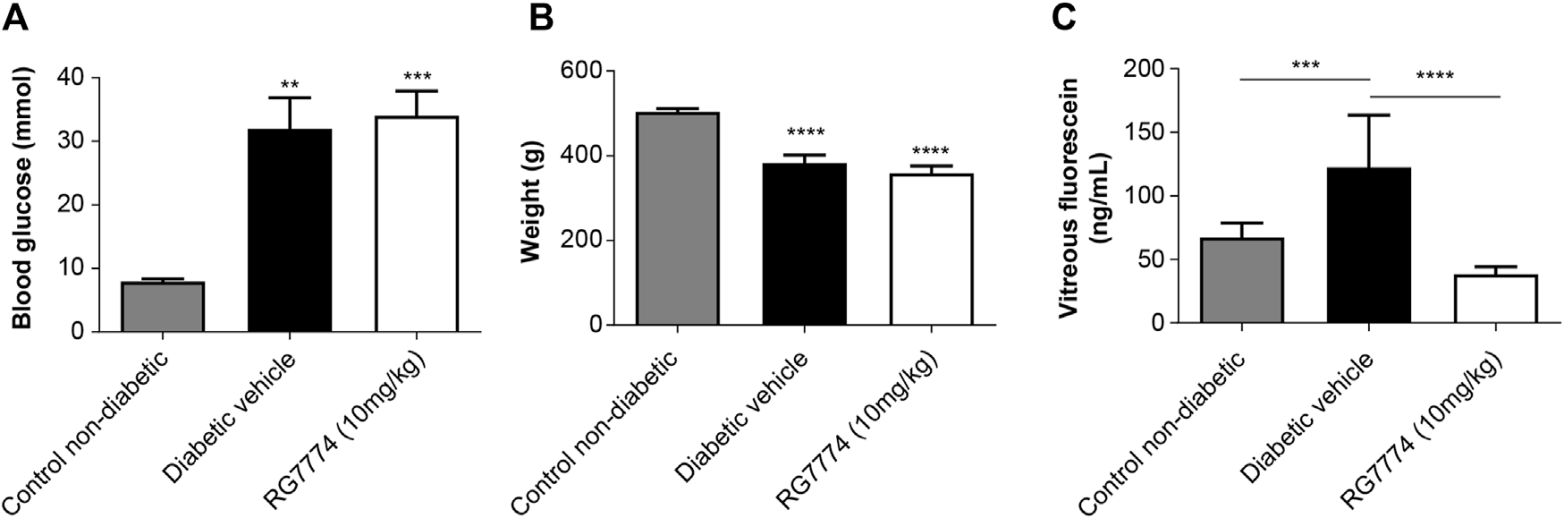
**(A)** Glucose levels (n = 10 animals for non-diabetic group, n = 7 animals for diabetic control group, and n = 9 animals for RG7774 10 mg/kg daily group) and **(B)** Body weights (n = 10 animals for non-diabetic group, n = 7 animals for diabetic control group, and n = 9 for RG7774 10 mg/kg daily group) 6 weeks after streptozotocin (STZ) injection in nondiabetic rats and diabetic rats treated with vehicle control or RG7774 10 mg/kg daily via oral gavage for 5 weeks. **(C)** Levels of vitreous fluorescein (n = 16 eyes for non-diabetic group, n = 12 eyes for diabetic control group, and n = 13 eyes for RG7774 10 mg/kg daily group) 6 weeks after STZ injection and 60 min after fluorescein injection in nondiabetic rats and diabetic rats treated with vehicle control or RG7774 10 mg/kg. Levels of fluorescein were normalized to levels in plasma samples taken 1 h after fluorescein injection. Data are shown as mean ± standard error of the mean (SEM) with n = 7–10 animals per group **(A,B)** or 12–16 eyes per group **(C)**. *p < 0.05, **p < 0.01, ***p < 0.001, and ****p < 0.0001 vs nondiabetic rats based on 1-way analysis of variance (ANOVA) **(A,B)** or 2-way ANOVA **(C)**, followed by Newman–Keuls *post hoc* analysis.

Levels of blood glucose were significantly higher in rats with STZ-induced diabetes treated with RG7774 or vehicle control (34.1 and 32.0 mmol, respectively) compared to nondiabetic controls (8.0 mmol) (*p* < 0.01 and < 0.001, respectively) (Figure 7A). Hyperglycemic rats treated with RG7774 or vehicle control weighed significantly less than untreated nondiabetic rats (359.3 and 383.3 g vs 504.6 g; *p* < 0.0001 for both) (Figure 7B). Fluorophotometry studies conducted 6 weeks after STZ injection and 10 min after fluorescein injection showed significantly more vitreous fluorescein in diabetic rats treated with vehicle control (93.5 ng/mL) than in untreated nondiabetic rats and diabetic rats treated with RG7774 [43.7 ng/mL (*p* < 0.001) and 25.6 ng/mL (*p* < 0.0001), respectively] (Figure 7C).

### 3.9 Effects of RG7774 in rats with laser-induced CNV

**FIGURE 8:**
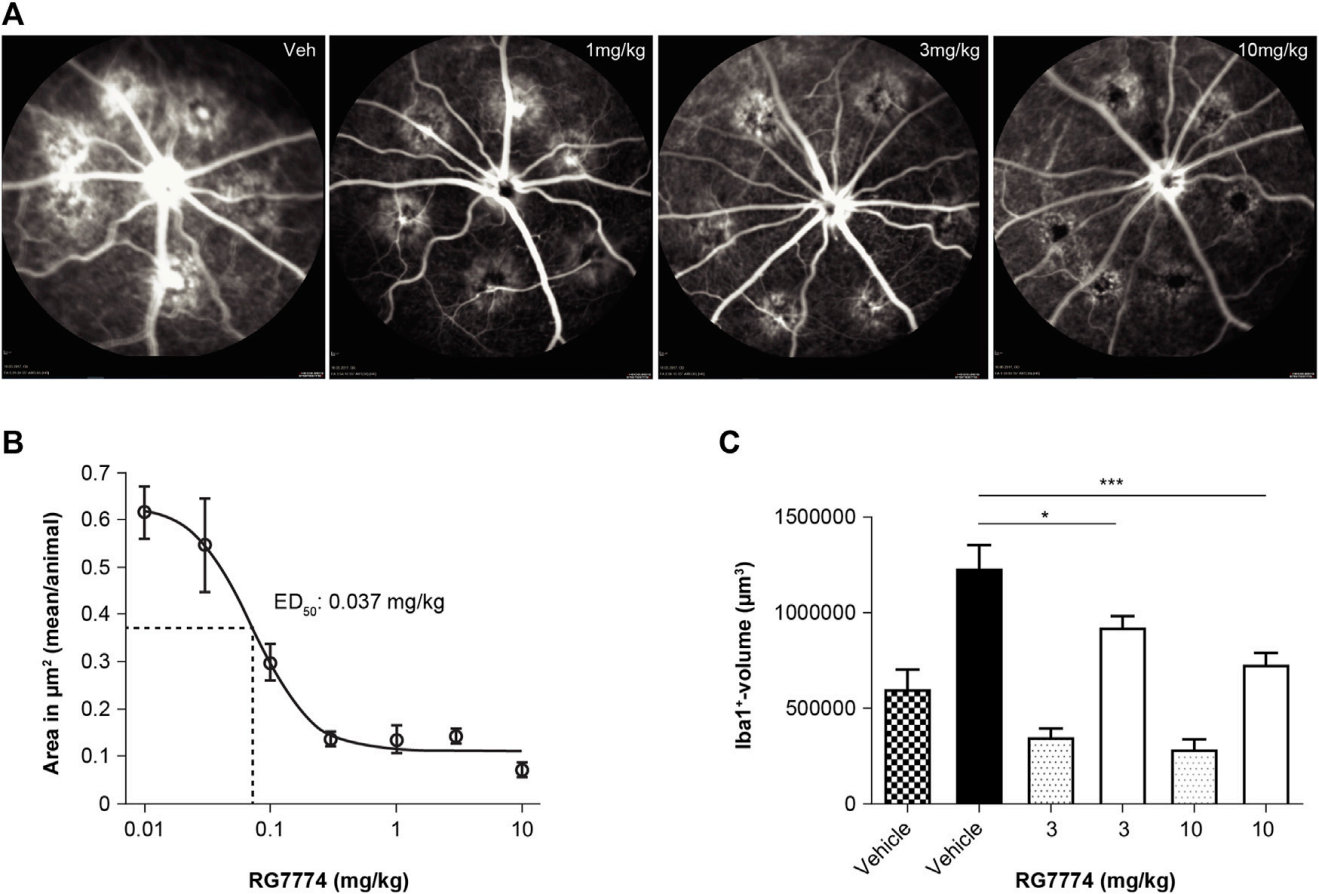
**(A)** Representative fluorescence angiography images (Heidelberg Engineering, Heidelberg Eye Explorer) of 6 laser-induced lesions per eye in rats treated with vehicle control (veh) or RG7774 1, 3, or 10 mg/kg via oral gavage, 1 day before and 7 days after laser injury. **(B)** Mean ± standard deviation (SD) lesion (hyperfluorescence) areas per eye determined by fluorescent angiography after intravenous (IV) injection of fluorescein in rats with laser-induced choroidal neovascularization (CNV) treated with vehicle control (n = 23 eyes) or RG7774 0.01 (n = 5 eyes), 0.03 (n = 5 eyes), 0.1 (n = 10 eyes), 0.3 (n = 5 eyes), 1.0 (n = 10 eyes), 3.0 (n = 13 eyes), 10 mg/kg (n = 13 eyes) via oral gavage. **(C)** Quantitative analysis of microglia migration toward laser-injured retinal regions in rats treated with vehicle control or RG7774 (3 and 10 mg/kg) via oral gavage. Data in **(C)** are shown as mean ± standard error of the mean (SEM) with 4, 5 and 5 stacks in non-lasered regions (patterned bars) and 24, 29 and 30 stacks in lasered regions for the vehicle, 3 and 10 mg/kg groups, respectively (solid bars). *p < 0.05; ***p < 0.001 based on 1-way analysis of covariance (ANOVA), followed by Newman–Keuls *post hoc* analysis. ED_50_: effective dose in 50% of animals.

Fluorescence angiography showed that the daily treatment of rats with RG7774 at the doses of 0.01–10 mg/kg PO 1 day before and 7 days after laser injury dose-dependently reduced lesion areas compared to vehicle control (Figures 8A,B), with an ED_50_ of 0.037 mg/kg. *Ex vivo* immunofluorescence of retinal flat mounts stained with Iba1 showed the migration of microglia toward the site of laser-induced injury. Microglia volumes in lasered regions increased by > 100% in all treatment groups compared to the respective distal nonlasered regions (Figure 8C). RG7774 3 and 10 mg/kg significantly and dose-dependently reduced microglia volumes in the lasered versus non-lasered regions of the same eye by 25% and 41%, respectively (Figure 8C).

## 4 Discussion

This preclinical proof-of-concept study characterizes the pharmacological and ADMET properties of the CB2R agonist, RG7774, and investigates its potential for managing the key pathological features associated with DR, both *in vitro* and in rodent models of retinal diseases. Competition binding studies using 2 radioligands, the CB1/2R-selective agonist [^3^H]-CP55940 and the newly developed CB2R-selective agonist [^3^H]-RO6753361 (Chicca et al., 2024), show that the binding affinities of RG7774 for recombinant human CB2R and endogenous human, rat, and mouse CB2R were high and similar across species, ranging from 31.9 ± 13.4 to 51.3 ± 16.2 nM. Cross-species similarities are consistent with the high level of amino acid conservation in the CB2R ligand binding pocket. Whereas Bmax was similar for endogenous CB2R in human, rat, and mouse cells, values were approximately 40-fold higher for CHO cells expressing recombinant human CB2R. Together, these results suggest that the binding affinity for RG7774 was unaffected by CB2R expression levels.

Modeling studies show that the *tert*-butyl moiety of RG7774 makes a large number of favorable contacts with the agonist-binding cavity of CB2R. This is in line with structure–activity relationship data, which show the replacement of the *tert*-butyl group by larger residues leads to a switch from agonism to inverse agonism (data on file). The high level of selectivity for CB2R over CB1R (>195-fold in competitive binding assays; > 3,600 in functional binding assays) can be rationalized by the docking poses for RG7774 on CB1R (PDB: 5XR8) (He et al., 2017) and CB2R (Figure 2B). Models indicate a steric clash between the triazole moiety of RG7774 and the CB1R residue, L193, which does not occur between RG7774 and the corresponding, less bulky, I110 residue on CB2R. This is important because the inadvertent activation of CB1R in the brain can lead to unwanted psychoactive side effects, making nonselective CB2R agonists unsuitable for clinical development (Pacher and Kunos, 2013; Dhopeshwarkar and Mackie, 2014; Tang et al., 2021). The low risk of off-target effects for RG7774, both in rodents and humans, was further demonstrated by *in vitro* binding assays for 78 nonendocannabinoid receptors, ion channels, and transporters and 11 endocannabinoid enzymes, including human 5-, 12-, or 15- LOX, MAGL, DAGL, EMT, FAAH and mouse MAGL, DAGL or FAAH.

Functional binding studies suggest that RG7774 is a potent, full agonist for recombinant human and mouse CB2R, with no significant effect on recombinant human CB1R or endogenous mouse CB1R. Whereas CB2R agonists in cAMP assays result in a decreased signal, CB2R-dependent β-arrestin recruitment results in a gain-of-signal that requires up to 100% receptor occupancy for a maximal effect. Consequently, the potency of RG7774 in CHO cells reported by β-arrestin recruitment assays (EC_50_ 99.69 ± 5.72 nM) was lower than that observed in cAMP assays (EC_50_: 2.81 ± 0.28 nM) and was closer to the Ki of 51.3 ± 16.2 nM observed in this cell line. Interestingly, CB2R activation did not inhibit forskolin-stimulated intracellular cAMP levels in Jurkat cells, suggesting a low CB2R-mediated G-protein activation efficiency. This is consistent with our current lack of understanding of CB2R-mediated downstream signaling in endogenous systems. However, a gain-of-signal pERK assay found that the potency of RG7774 in human Jurkat cells (EC_50_: 38.5 nM ± 15.0 nM) was almost identical to the binding affinity in this cell line (Ki: 31.9 ± 13.4 nM) and was slightly higher for RG7774-induced changes in cellular morphology, as measured via changes in impedance studies (EC_50_: 10.8 ± 4.2 nM). This suggests a functional effect for RG7774 mediated through the binding and activation of endogenous CB2R.

The PK, physicochemical, and ADMET profile for RG7774 suggest it is ideally suited for development as an oral treatment that targets the retina. The high degree of solubility (due to its tetrazole and 3-hydroxypyrrolidine moieties) and passive membrane permeability *in vitro* indicate that therapeutic doses of RG7774 in humans are likely to be completely absorbed. This observation is supported by simulations in a physiologically based pharmacokinetic (PBPK) model (GastroPlus; Simulations Plus, CA, United States) (data on file). The high degree of stability *in vitro* and the low-to-moderate clearance rates observed *in vitro* and *in vivo* are consistent with the moderate-to-high levels of bioavailability observed in mice and rats following systemic exposure. In addition, the low-to-moderate volume of distribution and distribution to the retina, choroid, and vitreous humor following IV or PO administration in rodents suggest that therapeutic doses of RG7774 are likely to reach the human eye. Oral self-administration of RG7774 presents a promising first-line treatment option for patients with unilateral or bilateral moderately severe to severe NPDR who maintain good vision and for whom IVT anti-VEGF therapy is typically deferred in clinical practice. This approach addresses the issue of low patient compliance associated with eye drops, offering a more convenient and potentially effective alternative.

The high levels of free RG7774 in human and rat plasma (16%–18%) were unexpected because endogenous CB2R ligands are fatty acid derivatives and cannabinoid-type agonists are generally reported to be tightly bound to protein (Soethoudt et al., 2017). Glutathione-stimulating hormone adduct formation and CYP inhibition assays indicate that RG7774 possesses an excellent early safety profile and does not interact with the human Ether-à-go-go-related gene (hERG) ion channel, which could lead to undesired cardiac effects (Warmke and Ganetzky, 1994; Sanguinetti and Tristani-Firouzi, 2006). Finally, RG7774 is a P-gp substrate (efflux extraction ratio 16.2 in mice and −2.5 in humans) with a high polar surface area (116 Å^2^), suggesting limited exposure in the CNS. Nevertheless, GLP toxicology studies in rats found that RG7774 is highly permeable with meaningful CNS penetration. Mean RG7774 brain:plasma ratios ranged from 0.0955 to 0.213 after oral administration in rats (data on file), and RG7774 concentrations in cynomolgus monkeys were approximately 10-fold lower in cerebrospinal fluid than in plasma (data on file).

IVT LPS injections cause EIU by damaging the blood barrier in the retina leading to increased vascular permeability with plasma protein leakage and leukocyte infiltration (Suzuki et al., 2011). In this study we used fluorophotometry and IVM to show that systemic treatment with RG7774 successfully prevented increases in retinal permeability and reduced leukocyte adhesion in rodents with LPS-induced EIU. Results are supported by a previous study in which a structurally similar CB2R agonist, RO6871304, reduced leukocyte-endothelial adhesion in LPS-injected wild type mice but had no effect in LPS-injected CB2R knockout mice, indicating that effects were mediated via CB2R (Porter et al., 2019). The immune-modulatory effects of CB2R are thought to occur via reduced levels of nuclear factor kappa light chain (NFκB) and activator protein-1 (AP-1) (transcription factor) expression, with subsequent reductions in downstream proinflammatory mediators, including TNFα, IL-1β, IL-6, IFNγ, CCL5, and CXCL2 (Toguri et al., 2014). Previous *in vitro* studies show that CB2R stimulation in monocytes downregulates the active forms of integrins, such as lymphocyte function-associated antigen 1 (LFA-1) and very late antigen 4 (VLA-4) (Rom et al., 2013). Consistent with this observation, CB2R agonism in a model of autoimmune uveoretinitis reduced leukocyte rolling and infiltration into the inflamed retina due to reductions in the expression of adhesion molecules, such as P-selectin glycoprotein and LFA-1 on T-cells (Xu et al., 2007). Leukocyte adhesion to the endothelium can also be regulated by endothelial intercellular adhesion molecule (ICAM) or vascular cell adhesion molecule (VCAM) expression (Granger et al., 2010). Interestingly, CB2R has also been shown to attenuate TNFα- and LPS-induced ICAM/VCAM expression in bovine brain endothelial cells (Ramirez et al., 2012) and in human retinal and coronary artery microvascular cells (Rajesh et al., 2007; Ontko et al., 2023), with subsequent reductions in leukocyte-endothelial cell interactions.

Previous studies in rats with STZ-induced type 1 diabetes show that antagonism of the leukocyte integrin, CD11b/CD18, using neutrophil inhibitory factor inhibits the formation of vascular lesions (Veenstra et al., 2013) and that genetic ablation of the CD18/ICAM interaction prevents leukostasis and reduces DR-associated retinal vascular pathology, including blood–retinal barrier breakdown, endothelial cell and pericyte loss, and the formation of acellular capillaries (Joussen et al., 2004). These data highlight the central and causal role of leukocyte adhesion in the pathogenesis of DR and suggest that leukostasis manifests as chronic, low-grade, subclinical inflammation leading to DR-related vascular lesions. More recently, CB2 activation using the agonists, AM1710, 5,6-EET-EA, and HU-308, has been shown to reduce the key features of DR, including leukocyte adhesion, vascular permeability, inflammation, and cell death, both *in vitro* and in rats with STZ-induced DR (Stark et al., 2021; Spyridakos et al., 2022; Ontko et al., 2023). Here we show that diabetes-induced vascular permeability was fully prevented in rats treated with oral RG7774, whereas blood glucose levels and body weight were unaffected. This suggests that the effects of RG7774 on retinal vascular permeability were not due to glycemia-lowering.

Modest levels of CB2R expression have been demonstrated in retinal microglia under basal conditions, with levels increasing after inflammatory insult (Borowska-Fielding et al., 2018). Similarly, microglial cell activation is induced by injury, infection, and inflammatory diseases, such as DR and age-related macular degeneration (Rashid et al., 2019). Upon activation, microglia acquire an amoeboid morphology and a highly migratory behavior that causes the cells to invade (particularly) the outer retinal nuclear layer which is commonly devoid of immune cells (Murenu et al., 2022). In this study, we used fluorescence angiography to show that the oral treatment of rats with RG7774 prior to and after retinal laser injury dose-dependently and potently reduced the lesion area. The average concentration of the free active epimer in plasma (1.05 nM) and the maximum concentration (12.5 nM) associated with the ED_50_ of 0.037 mg/kg in the CNV rat model were in the range of the human CB2R EC_50_ obtained from *in vitro* functional cellular assays (2.81 nM in the cAMP assay with recombinant CHO cells and 10.8 nM in the impedance assay with Jurkat cells).^1^ This suggests that the reduction in microglial migration was associated with reduced microglial activation. Prevention of laser-induced retinal leakage and microglial migration have previously been observed following the pharmacological inhibition of NF-kB (Hikage et al., 2021), which is in line with CB2R-mediated inhibition of NF-kB (Toguri et al., 2014). Further investigations are necessary to elucidate the mechanism of CB2R activation in the retinas of animals with type 2 diabetes, given the limitations of the current study. To date, the anti-inflammatory effects observed in the LPS-induced EIU model have been attributed to CB2R through genetic ablation or pharmacological inhibition. However, the reduction of RG7774-mediated permeability in the same *in vivo* model, the diabetic STZ model, and laser-induced CNV in rats lacks appropriate controls to confirm CB2R specificity. Furthermore, the incomplete understanding of the molecular mechanism of CB2R activation has hindered the demonstration of modulation of disease-relevant biomarkers, such as VEGF or ICAM, in relevant cell types like retinal endothelial cells or microglia. This area is currently the subject of further studies in our laboratories.

In conclusion, RG7774 is a novel, highly selective, and orally bioavailable CB2R agonist, with an acceptable systemic and ocular PK profile in rodents. Beneficial effects on retinal vascular permeability, leukocyte adhesion, and ocular inflammation in rodent models of retinal disease support the development of RG7774 as a potential oral treatment for retinal diseases with similar pathophysiologies.

## Scope statement

First-line pharmacotherapy for diabetic retinopathy (DR) includes regular intravitreal injections with anti-vascular endothelial growth factor (anti-VEGF) therapies. Although effective, intravitreal anti-VEGF injections are highly invasive, and are associated with a high degree of treatment burden, patient anxiety, and an infrequent but serious risk of intraocular inflammation. This preclinical proof-of-concept study shows that RG7774 is a novel, highly selective, and orally bioavailable cannabinoid 2 receptor (CB2R) agonist, with a favorable systemic and ocular pharmacokinetic profile, and beneficial effects on vascular permeability, leukocyte adhesion, and ocular inflammation (key pathological features of DR) in animal models of retinal disease. Results support the development of RG7774 as a potential noninvasive treatment for DR and other retinal diseases with similar pathophysiology.

## Data Availability

The original contributions presented in the study are included in the article/Supplementary Material, further inquiries can be directed to the corresponding author.

## Footnotes

1 Previously, RG7774 epimerization *in vitro* and *in vivo* was observed in rats (but not in mouse and human). Epimerization is thought to occur via CYP-mediated oxidation of RG7774 to generate the ketone form, followed by reduction to either regenerate RG7774 (S-enantiomer) or the R-enantiomer. An S/R-enantiomer ratio of 2 was assumed based on PK measurements obtained using a chiral bioanalytical assay (data on file).
