# Supplementary material for: RG7774 (Vicasinabin), an orally bioavailable cannabinoid receptor 2 (CB2R) agonist, decreases retinal vascular permeability, leukocyte adhesion, and ocular inflammation in animal models
Source: Front Pharmacol. 2024 Jul 12;15:1426446. doi: 10.3389/fphar.2024.1426446 (PMC11272598; doi:10.3389/fphar.2024.1426446)
Supplement: Supplementary file 1 [file Table1.docx]

***Supplementary materials***

***RG7774 (Vicasinabin), an orally bioavailable cannabinoid receptor 2 (CB2R) agonist, decreases retinal vascular permeability, leukocyte adhesion and ocular inflammation in animal models***

Uwe Grether, Richard H. Foxton, Sabine Gruener, Claudia Korn, Atsushi Kimbara, Anja Osterwald, Elisabeth Zirwes, Sabine Uhles, Janina Thoele, Nadine Colé, Mark Rogers-Evans, Stephan Roever, Matthias Nettekoven, Rainer E. Martin, Jean-Michel Adam, Jürgen Fingerle, Caterina Bissantz, Wolfgang Guba, André Alker, Anna M. Szczesniak, Ross F. Porter, Tom J. Toguri, Franco Revelant, Agnès Poirier, Camille Perret, Lotte Winther, Antonello Caruso, Filomena Fezza, Mauro Maccarrone, Melanie E.M. Kelly, Sascha Fauser, and Christoph Ullmer^*^

*** Correspondence:** Christoph Ullmer, christoph.ullmer@roche.com

1. **Supplementary Methods**

**S1.** **Synthesis of (*S*)-1-(5-*tert*-butyl-3-[(1-methyl-1*H*-tetrazol-5-yl)methyl]-3*H*-[1,2,3]triazolo[4,5-d]pyrimidin-7-yl)pyrrolidin-3-ol RG7774.**

(**A**) Sodium azide (4.34 g, 66.0 mmol, Eq: 1.05) was charged in the reactor followed by dimethylsulfoxide (DMSO; 44.0 g, 40 mL) and Hünig’s base (829 mg, 1.12 mL, 6.29 mmol, Eq: 0.1). The suspension was stirred for 10 min at 25°C. (Chloromethyl)benzene (8 g, 7.29 mL, 62.9 mmol, Eq: 1.00; CAS number 100-44-7) was added dropwise over 1 h at 25°C. After 3 h at 25°C, water (1.6 g, 1.6 mL) was added, the reaction mixture was stirred for 30 min, filtered, and the residue was washed with DMSO (17.6 g, 16.0 mL). The obtained benzyl azide **2** solution was used directly in the subsequent cycloaddition step. (**B**) A reactor was charged with DMSO (17.6 g, 16.0 mL), followed by 32% aqueous NaOH (7.86 g, 5.82 mL, 62.9 mmol, Eq: 1.0) and water (5.00 g, 5.00 mL). A solution of 2-cyanoacetamide (7.93 g, 94.3 mmol, Eq: 1.50; CAS number 107-91-5) in DMSO (17.6 g, 16.0 mL) was added dropwise over 15 min at 25°C. The previously prepared benzyl azide solution **2** was added dropwise over 4 h at 25°C and the reaction was stirred overnight at 25°C. Water (120 g, 120 mL) was added dropwise over 30 min at 25°C and the resulting suspension was cooled over 30 min to 0°C, stirred at 0°C for 30 min and filtered. The filter cake was washed with water (40.0 g, 40.0 mL) and dried at 50°C / 5 mbar to give the title compound **3** (12.6 g, 58 mmol, 92% over 2 steps) as white solid. (**C**) 5-Amino-1-benzyl-1*H*-1,2,3-triazole-4-carboxamide **3** (150 g, 691 mmol, Eq: 1.00) was suspended in *N*,*N*-dimethylacetamide (512 g, 550 mL). Pyridine (82.1 g, 83.5 mL, 1.04 mol, Eq: 1.5) was added followed by pivaloyl chloride (126 g, 129 mL, 1.04 mol, Eq: 1.5) and the mixture was heated to 80°C. After 1.5 h, acylation to 1-benzyl-5-(2,2-dimethylpropanoylamino)triazole-4-carboxamide **4** was completed (LC-MS). KHCO_3_ (347 g, 3.45 mol, Eq: 5.00) was added and the suspension was heated to 155°C for 18.5 h to convert carboxamide **4** to the title compound. The reaction mixture was cooled to room temperature (RT) and water (3.48 kg, 3.48 L) was added dropwise within 30 min. The light yellow suspension was stirred for 30 min at RT and then for 2 h at 0°C before filtering. The filter cake was washed with cold (0–5°C) water (600 g, 600 mL) and dried at 50°C / 5 mbar to give the title compound **5** (161.3 g, 569 mmol, 82% over 2 steps) as off-white powder. (**D**) DMF over molecular sieves (105 g, 110 mL, 1.43 mol, Eq: 2.56) was charged in the reactor followed by dichloromethane (1.46 kg, 1.1 L). The solution was heated to 35°C. Oxalylchloride (144 g, 97.6 mL, 1.11 mol, Eq: 2) was added over 1 h. After 45 min, a fine suspension of 3-benzyl-5-*tert*-butyl-4*H*-triazolo[4,5-d]pyrimidin-7-one **5** (161 g, 557 mmol, Eq: 1.00) in a mixture of dichloromethane (877 g, 662 mL) and DMF (41.8 g, 44.1 mL) was added over 20 min. After 3 h the reaction mixture was cooled to RT and slowly added to a cold (0–5°C) half-saturated aqueous NaHCO_3_ (1.76 L). The organic phase was separated and washed again with half-saturated NaHCO_3_ (662 mL) followed by water (662 g, 662 mL). Then the organic phase was dried over MgSO_4_ and concentrated under reduced pressure at 50°C / down to 10 mbar to give a crude oil which crystallized upon standing. The crude 3-benzyl-5-*tert*-butyl-7-chloro-triazolo[4,5-d]pyrimidine **6** (192.2 g) was introduced in the next step without further purification. 3-Benzyl-5-*tert*-butyl-7-chloro-3*H*-[1,2,3]triazolo[4,5-d]pyrimidine **6** (192.2 g, 548 mmol, Eq: 1.00) was charged in the reactor followed by acetonitrile (780 g, 1.0 L) and *N*-ethyldiisopropylamine (108 g, 143 mL, 822 mmol, Eq: 1.5). (*S*)-Pyrrolidin-3-ol (54.1 g, 51.6 mL, 603 mmol, Eq: 1.1; CAS number 100243-39-8) was added dropwise over 30 min keeping the temperature between 20°C and < 30°C. After 2 h at 25°C the reaction mixture was transferred into a 3 L round bottom flask containing toluene (865 g, 1.0 L) and concentrated on a rotary evaporator to switch the solvent to toluene. The toluene solution was washed with a 10% aqueous citric acid solution (1.0 L) and the aqueous phase was separated and extracted with toluene (434 g, 500 mL). The organic phases were washed sequentially with half-saturated aqueous NaHCO_3_ (500 mL) and half-saturated aqueous NaCl (500 mL). The organic layers were combined, dried over MgSO_4_ and concentrated at 45°C to approximately 500 mL. Heptane (684 g, 1.0 L) was added, the mixture was stirred and, after 10 min the product started to crystallize. The white suspension was stirred for 2 h at RT and filtered. The white filter cake was washed with heptane (274 g, 400 mL) and dried at 45°C / 5 mbar to give the title compound (186.9 g, 530 mmol, 95% over 2 steps) as a white powder. (**E**) (*S*)-1-(3-Benzyl-5-*tert*-butyl-3*H*-[1,2,3]triazolo[4,5-d]pyrimidin-7-yl)pyrrolidin-3-ol **7** (176 g, 494 mmol, Eq: 1.00) was dissolved in methanol (2.09 kg, 2.64 L). 1.25 M HCl in methanol (396 mL, 494 mmol, Eq: 1.00) was added followed by 10% Pd/C (34.7 g, 32.6 mmol, Eq: 0.066). The reaction mixture was hydrogenated for 20 h at 60°C / 1 bar, cooled, evacuated, purged, and filtered. The light yellow solution was concentrated at 50°C to approximately 1 L. Toluene (1.3 kg, 1.5 L) was added and the solution was concentrated at 50°C / 150 mbar to approximately 1.2 kg to remove most of the methanol upon which the product started to crystallize. The white suspension was cooled to RT, stirred for 1 h and filtered. The filter cake was washed with toluene and dried at 50°C / 5 mbar to give the title compound (140.5 g, quant.) as a white solid. From microanalysis data, the structure would be consistent with a hemi hydrochloride. (**F**) (*S*)-1-(5-*tert*-Butyl-3*H*-[1,2,3]triazolo[4,5-d]pyrimidin-7-yl)pyrrolidin-3-ol x 0.5 HCl **8** (5 g, 17.8 mmol) was dissolved in DMF (23.7 g, 25.0 mL). DBU (6.43 g, 6.37 mL) was added dropwise over 5–10 min. A solution of 5-(chloromethyl)-1-methyl-1*H*-tetrazole (3.33 g, 25.1 mmol; CAS number 57235-84-4) in DMF (23.7 g, 25.0 mL) was added dropwise over 35 min at RT. The orange solution was stirred for 2 h. The reaction mixture was added to 25% aqueous NH4Cl (100 mL). MTBE (148 g, 200 mL) was added. The aqueous phase was separated and extracted with MTBE (148 g, 200 mL). The organic phases were washed sequentially with water (100 g, 100 mL) and half-saturated NaCl (100 mL). Then the organic phases were combined, dried over MgSO_4_ and evaporated at 45°C / down to 10 mbar to give 5.75 g of crude product as a white foam. A portion (3.6 g) of the crude product was purified by preparative SFC (column: Kromasil 60 SIL, 5 µm, 21.2 x 250 mm, 80% CO_2_/20% MeOH, 40°C) to give 1.98 g of the title compound. Crystallization: 1.3 g of the product was crystallized from *i*PrOAc/Heptane (1:1) to give RG7774 (1.2 g, 3.35 mmol, 19%) as white powder. Yields refer to purified compounds.

**S2. Physicochemical and ADMET studies**

**S2.1 Kinetic lyophilization solubility assay (LYSA)**

RG7774 samples were prepared in duplicate from 10 mM DMSO stock solutions. DMSO was evaporated for 1 h using a centrifugal vacuum evaporator (Genevac Technologies, UK) and the residue was dissolved in 0.05 M PBS (pH 6.5), stirred for 1 h, and shaken for 2 h. Solutions were filtered 12 h later using a microtiter filter plate (MSDV N65; Merck Millipore, MA, USA). The filtrate and its 1:10 dilution were analyzed by direct ultraviolet (UV) measurement or high-performance liquid chromatography with UV spectroscopy (HPLC-UV). A 4-point calibration curve was prepared from the 10 mM DMSO stock solutions and used to determine the kinetic solubility of the test compounds. The measurement range for a compound with molecular weight 500 was 0 to 666 μg/mL.

**S2.2 Thermodynamic solubility assay (THESA)** [1]

RG7774 (8.6 mg per mL solvent/vehicle) was stirred in HPLC vials (9 × 12 × 32 mm; Waters UK) at 350 rpm for 15 h [2]. The solvent was 0.05 M aqueous phosphate buffer and vehicles were fasted or fed simulated gastrointestinal fluid (FaSSIF or FeSSIF). The presence of solid particles was determined by analyzing 10 μL samples under a microscope. If the active pharmacological ingredient (API) was completely dissolved, more solid API was added and stirred for another 15 h. This step was repeated for up to 96 h or until residual solid particles could be detected. Samples (0.5 mL) were transferred to Eppendorf Ultrafree filter tubes (Filter: PVDF 0.22 μm) and centrifuged at 14,500 rpm for 10 min. The filtrates were diluted in ethanol and analyzed by ultra-pure liquid chromatography.

**S2.3 Aqueous stability assay (ASTA)**

Aqueous solutions of RG7774 were prepared at 5 different pHs (pH 1-10) from DMSO stock solutions, added to incubation plates and shaken for 10 min at 37 °C. Solutions were transferred to a filter plate (Millipore MSGVN2250, pore size 0.22 μm) and filtered into V-bottom plates (ABGene, AB-0800) prior to heat-sealing. The procedure was repeated, increasing the 37 °C incubation time by 2 h. Samples were analyzed by HPLC at 0 and 2 h. A compound was classified as unstable if < 90% of the initial concentration was detected after 2 h.

**S2.4 Parallel artificial membrane permeability assay (PAMPA)**

A 96-well microtiter plate was filled with aqueous buffer (typically 0.05 M Tris, pH 7.4, or 0.05 M phosphate, pH 6.5) and covered with a filter plate in a sandwich construction. The hydrophobic filter material (Durapore/Millipore; pore size 0.22–0.45 μm) covering the first 48 wells (sample) was impregnated with 1%–20% lecithin dissolved in dodecane, hexadecane, and 1,9-decadiene. The filter cover for the remaining 48 wells (reference) was wetted with 4–5 μL of a 50% (*v*/*v*) methanol/buffer solution. The transfer of RG7774 and reference substances from the donor compartment, through the lipid-infused artificial membrane, into the acceptor compartment was initiated by transferring 100–200 μL of a 250 or 500 μM stock solution to filter plates covering the sample and reference wells, respectively. The maximum DMSO content of the stock solutions was 5%. The permeation coefficient (*P_eff_*) was determined as previously described by Kansy et al. 1998 [3].

**S2.5 Microsomal clearance**

Commercially-available ultra-pooled microsome preparations from human (150 different donors) (BD UltraPool HLM 150, Beckton Dickinson, Japan) or mouse liver tissues (Beckton Dickinson) [4] were incubated in 96-deep-well plates containing 0.1 M phosphate buffer (pH 7.4) at 37 °C on a TECAN liquid handling system equipped with Teleshake shakers and a warming device (Tecan Group Ltd., Switzerland). The NADPH regenerating system consisted of 30 mM glucose-6-phosphate disodium salt hydrate, 10 mM NADP, 30 mM MgCl_2_ × 6 H_2_O, and 5 mg/mL glucose-6-phosphate dehydrogenase (Roche Diagnostics, IN, USA) in 0.1 M potassium phosphate buffer (pH 7.4). The microsome mixture (0.5 mg/mL) was incubated with 1 μM RG7774 in 96-well plates at 37 °C. After 1, 3, 6, 9, 15, 25, 35, and 45 min, aliquots of the incubations (40 μL) were removed and reactions were quenched with 1:3 (*v*/*v*) acetonitrile containing internal standards. Samples were cooled, centrifuged, and analyzed by liquid chromatography-mass spectroscopy (LC-MS)/mass spectroscopy (MS). Log peak area ratios (test compound peak area/internal standard peak area) were plotted against incubation time with a linear fit. The calculated slope was used to determine the intrinsic clearance: CL_int_ (μL/min/mg protein) = –slope (min–1) × 1000/protein concentration, mg/mL.

**S2.6 Hepatocyte clearance**

For animals, hepatocyte suspension cultures were either freshly prepared by liver perfusion or from cryopreserved hepatocyte batches (BioIVT, NY, USA). For humans, commercially-available, pooled (5–20 donors), cryopreserved human hepatocytes from nontransplantable liver tissues were used (BioIVT) [5]. Suspension cultures were incubated in Nunc U96 PP-0.5 mL plates (Nunc Natural, 267245) in a Thermo Forma incubator (Fischer Scientific, Wohlen, Switzerland) equipped with Variomag® Teleshake shakers (Sterico, Wangen, Switzerland) to maintain cell dispersion. The cell culture medium was William’s media supplemented with glutamine, antibiotics, insulin, dexamethasone, and 10% FCS. RG7774 (1 μM) were incubated in 96-well plates containing suspension cultures (1 × 10^6^ hepatocytes/mL [~1 mg/mL protein concentration]), with each well containing a total volume of 100 μL. Plates were shaken at 900 rpm for up to 2 h in 5% CO_2_ at 37 °C. Reactions were quenched after 3, 6, 10, 20, 40, 60, and 120 min with 200 μL methanol containing an internal standard. Samples were cooled, centrifuged, and analyzed by LC-MS/MS. Log peak area ratios (test compound peak area/internal standard peak area) or concentrations were plotted against incubation time using a linear fit to the data with emphasis on the initial rate of compound disappearance. The slope of the fit was used to calculate the intrinsic clearance: CL_int_ (μL/min/1 × 10^6^ cells) = –slope (min^–1^) × 1000/(1 × 10^6^ cells).

**S2.7 Plasma protein binding**

Pooled and frozen plasma from selected species were obtained from commercial suppliers (human HMPLEDTA and mouse MSEPLEDTA3-C57; BioreclamationIV, NY, USA) [6, 7]. Unbound RG7774 was identified using a 96-well, 150 μL, half-cell capacity Teflon equilibrium dialysis device with a 12–14 kDa molecular weight cut-off membrane (HT-Dialysis; Gales Ferry, CT, USA) to minimize nonspecific binding. RG7774 and positive control (diazepam) were tested with an initial total concentration of 1000 nM (cassettes of 2 to 5 test items). Equal volumes of matrix samples containing RG7774 or diazepam and blank (Soerensen) dialysis buffer were loaded into the opposite compartments of each well. Biological matrix and phosphate buffer were adjusted to pH 7.4 on the day of the experiment. The dialysis block was sealed and incubated at 37 °C in 5% CO_2_ for 5 h. The seal was removed and matrix and buffer from each dialysis were sampled and analyzed by LC-MS/MS. All protein binding determinations were performed in triplicate. Membrane integrity was tested in the HT-Dialysis device by determining the unbound fraction values for diazepam in each well. At equilibrium, the unbound drug concentration in the biological matrix compartment of the equilibrium dialysis apparatus is the same as the concentration of the compound in the buffer compartment. Thus, the percent unbound fraction was calculated as follows: % fraction unbound = 100 × buffer concentration after dialysis/matrix concentration after dialysis. Device recovery was checked by measuring the concentrations of compound in the matrix before dialysis and calculating the percent recovery (mass balance). Recovery ranging from 80%–120% was required for data acceptance.

**S2.8 Permeability-glycoprotein (p-gp) assay**

The P-gp assay was conducted as previously described [8]. Briefly, transfected porcine kidney epithelial (LLC-PK1) cells expressing human or mouse P-gp were cultured on 96-well semi-permeable filter membrane plates (Millipore, Darmstadt, Germany). LLC-PK1 cells were provided by Dr A. Schinkel from The Netherlands Cancer Institute (Amsterdam, The Netherlands). Cells formed a polarized monolayer with tight junctions that acted as a barrier between apical and basolateral compartments. P-gp was expressed in the apical-facing membrane of the monolayer (tightness confirmed using Lucifer yellow). To determine the unidirectional permeability (Papp) of RG7774, samples were added separately to the apical (for A > B Papp) and basolateral (for B > A Papp) sides of the cell monolayer (i.e., donor compartments), and RG7774 movement into the respective receiver compartments was measured by LC-MS/MS over a 3 h incubation at 37 °C. The effect of P-gp was measured by expressing the efflux ratio of the unidirectional A > B and B > A Papp values. The mean permeability (A > B and B > A Papp) was determined in the absence of P-gp via the addition of the selective P-gp inhibitor, zosuquidar.

**S2.9 Cytochrome P450 (CYP) 3A4, 2C19, 2C8, 2C9, 2D6, and 1A2 inhibition assay**

CYP assays were conducted as previously described [9] to determine the risk of drug-drug interactions between test compounds and drugs known to be metabolized via different CYPs. RG7774 was incubated at a range of concentrations with the following components: pooled human liver microsomes, CYP probe substrate around the reported K_m_, and NADPH (final concentration: 1 mM) in 100 mM sodium phosphate buffer (pH 7.4). Conditions were optimized to achieve the linear metabolic rate for the probe substrate reactions. Samples were analyzed by LC-MS/MS. Assays generate two endpoints: IC_50_ (μM) and percent inhibition at the highest acceptable test concentration (typically 50 μM; lower if highest concentration data are rejected due to insolubility).

**S2.10 Glutathione-stimulating hormone (GSH) adduct formation in human liver Microsomes**

GSH adduct formation assays were conducted as previously described [10]. Briefly, RG7774 was incubated with human liver microsomes to form reactive metabolites, and glutathione was added as a nucleophile to convert the reactive metabolites into stable conjugates that could be analyzed by MS. The formation of reactive metabolites suggests a test compound might trigger drug-induced liver injury and drug-induced hypersensitivity reactions in patients.

## S3. Competitive binding assay in mouse brain

Mouse brain was resuspended in 2 mM Tris–EDTA, 320 mM sucrose, 5 mM MgCl_2_ (pH 7.4), homogenized using a glass potter, and centrifuged 3 times, each at 1000 g for 10 min. The initial pellet was discarded, the supernatant was centrifuged at 18000 g for 30 min, and the final pellet was resuspended in assay buffer (50 mM Tris-HCl, 2 mM Tris–EDTA, 3 mM MgCl_2_, pH 7.4) as previously described [11]. Membrane fractions (50–100 µg) were used in rapid filtration assays with the radiolabeled agonist, [^3^H]-CP55940, 400 pM (PerkinElmer Life Sciences, oston, MA, USA) bound to 284 ± 26 fmol cannabinoid 1 receptor (CB1R) binding sites per mg of protein (CTRL), as described in Section 2.2.2 of the main manuscript. Non-specific binding was determined using 1 µM of “cold” control agonist (CP55940); >95% of the total binding signal was specific.

## S4. Soybean 15-lipoxygenase (LOX) assay

The inhibitory effect of RG7774 on soybean 15-LOX activity was determined using the Lipoxygenase Inhibitor Screening Assay Kit (Cayman Chemical Company, MI, USA). 15-LOX was incubated with linoleic acid (100 µM) at RT for 30 min and the resulting hydroperoxides were measured using plate-based colorimetry (490-500 nm; VICTOR X3, PerkinElmer). The effect of RG7774 (preincubated for 15 min) at 10 µM on 15-LOX was ascertained by adding the compounds directly to the incubation medium (0.1 M Tris-HCl, pH 7.4) in a final volume of 100 µL. The LOX inhibitor, NDGA, 100 µM (supplied with the assay kit) was used as a positive control.

## S5. Human 5-, 12-, and 15-LOX assay

The inhibitory effects of different concentrations of RG7774 (0.1-500 µM) on the activities of human recombinant 5-LOX (Cayman Chemical Company #60402), 15-LOX (Cayman Chemical Company #10011263), and human platelet-derived 12-LOX were determined using the Biovision Lipoxygenase Activity Assay Kit (#BVN-K978-100). This kit converts the LOX substrate to an intermediate that reacted with the probe, generating a fluorescent product. The effect of RG7774 (preincubated for 15 min) at 10 µM on LOXs (5-LOX: 0.5 or 1.2 µg; 12‑LOX: 20 µg; 15-LOX: 0.23 or 0.3 µg) was ascertained by adding the compounds directly to the incubation medium and the fluorescent signal, recorded at excitation/emission 500/536 nm at 30 sec intervals for 30-40 min, was directly proportional to LOX activity. The LOX inhibitor, NDGA (100 µM) (included in the kit) was used as a positive control. The percentage of human 5-, 12-, and 15-LOX activity (control) was estimated from the amount of oxidized probe in the reaction using a standard curve.

## S6. Mouse and human ****monoacylglycerol lipase****(MAGL) assay

Mouse brain or human U937 cell extracts (ATCC, Manassas, VI, USA) were resuspended in 50 mM NaPO_4_ buffer containing 320 mM sucrose (pH 8.0), homogenized at 4°C using a glass potter, and centrifuged at 4°C sequentially at 800 g (10 min), 10,000 g (30 min), and 100,000 g (60 min). The final supernatant was incubated with [^3^H]-2-OG (10 µM) (ARC, MO, USA) at 37°C for 30 min as previously described [12], and reactions were stopped with a 2:1 (v/v) mixture of chloroform/methanol. [^3^H]-glycerol release in the aqueous phase was measured by scintillation counting. Mouse supernatant (100 µg) was preincubated for 15 min at RT with RG7774 (10 µM) in the incubation medium (Tris-HCl [50 mM], pH 7.4). Human supernatant (100 µg) was preincubated for 15 min at RT with 8 different concentrations of RG7774 (0.1-500 μM) in the incubation medium. Experiments were repeated using the selective MAGL inhibitor, JZL-184 (100 nM), as a positive control.

## S7. Mouse and human ****diacylglycerol lipase****(DAGL) assay

Mouse brain or human HaCaT cells (CLS Cell Lines Service, GmbH, Eppelheim, Germany) were homogenized in 50 mM Tris-HCl (pH 7.4) using a glass potter, and homogenates were centrifuged at 4°C sequentially at 800 g (10 min) and 10,000 g (30 min). The final pellet (80–200 µg) was resuspended in 50 mM Tris-HCl (pH 7.4) containing 10 mM CaCl_2_ and incubated at 37°C for 30 min with *sn*-1[^14^C]-stearoyl-2-arachidonoyl-glycerol (100 µM) (ARC, MO, USA). Reactions were stopped by adding chloroform/methanol (2:1, v/v), and the organic phase was dried and fractionated by thin-layer chromatography on silica gel, as previously reported [12]. Levels of 1[^14^C]-stearoyl acid radioactivity were determined by cutting the appropriate band from the gel and analyzing using a β-scintillation counter. Mouse cells were preincubated for 15 min with RG7774 (10 µM) and added to the incubation medium. Human cells were preincubated for 15 min with 8 different concentrations of RG7774 (0.1-500 μM) and added to the incubation medium. Control experiments were carried out in the presence of the selective DAGL inhibitor, D053 (10 µM).

**S8. Human endocannabinoid membrane transporter (EMT) activity**

HaCaT cells were seeded (350,000–400,000 cell/well) in triplicate in 12-well plates and incubated for 15 min in PBS at 37°C with [^3^H]-N-arachidonoylethanolamine  ([^3^H]-AEA) (400 nM) (ARC, MO, USA), as previously reported [13]. Experiments were repeated at 4°C in order to subtract passive diffusion from EMT-mediated uptake. RG7774 (10 µM) was preincubated with labeled HaCaT cells for 15 min before adding directly to the incubation medium (PBS). The selective EMT inhibitor, OMDM-1 (40 µM), was used as positive control.

## S9. Mouse and human fatty acid amide****hydrolase (****FAAH) assay

Mouse brain was homogenized in 50 mM Tris-HCl (pH 7.4) using a glass potter, centrifuged at 1000 g for 10 min, and the pellet was discarded. The supernatant (50 µg) was incubated with [^14^C]-AEA (10 µM) (ARC, MO, USA) at 37°C for 15 min (Tris-HCl, pH 9.0) and reactions were stopped with a 2:1 (v/v) mixture of chloroform/methanol. [^14^C]-ethanolamine release in the aqueous phase was measured as previously reported [12]. Mouse supernatants (50 µg) were preincubated with RG7774 (10 µM) for 15 min and added directly to the incubation medium. Purified human FAAH (1.7 µg; Cayman Chemical Company, MI, USA) was incubated with [^14^C]-AEA (10 µM) (ARC, MO, USA) at 37°C for 15 min (pH 9.0), as previously described [12]. RG7774 was preincubated for 15 min with 8 different concentrations of RG7774 (0.1-500 μM) and added directly to the incubation medium (250 μL). Control experiments was carried out in the presence of the FAAH inhibitor, URB-597 (100 nM).

## S10. Mouse cyclic adenosine monophosphate (cAMP) assay

The effect of RG7774 on the forskolin-stimulated accumulation of cAMP was determined using the LANCE ULTRAcAMP kit (PerkinElmer Life Sciences, MA, USA), according to the manufacturer’s instructions. Briefly, N18TG2 cells preincubated for 30 min with 1-methyl-3-isobutylxanthine (1 mM) were added to 0.5 µM forskolin preincubated with or without RG7774 for 15 min. Reactions were incubated for 30 min at 37°C and Time-resolved fluorescence was measured using a Victor V Multilabel counter (PerkinElmer Life Sciences). Data were transformed into cAMP concentrations using a standard curve.

1. **Supplementary Tables**

**Supplementary Table S1.** Crystallographic information on RG7774 data collection and structure refinement.

| Formula | C15H22N10O |
| --- | --- |
| Crystal class | Orthorhombic |
| Space group | P2(1)2(1)2(1) |
| a (Å) | 9.11460(10) |
| b (Å) | 17.6394(2) |
| c (Å) | 21.82910(10) |
| α (°) | 90 |
| β (°) | 90 |
| γ (°) | 90 |
| Volume (Å3) | 3509.60 |
| Z | 8 |
| Crystal size (mm) | 0.30 x 0.20 x 0.15 |
| Radiation type | Copper |
| Wavelength (Å) | 1.54184 |
| Density (mg/cm^3^) | 1.357 |
| Temperature (K) | 100 (2) |
| Reflections measured | 127019 |
| Independent reflections | 6777 |
| R1 [I>2sigma(I)] | 0.0285 |
| wR2 [I>2sigma(I)] | 0.0768 |
| R1 (all data) | 0.0289 |
| wR2 (all data) | 0.0772 |
| Numbers of parameters | 485 |
| Goodness of fit | 1.047 |
| CCDC | 2281444 |
| Absolute structure parameter | 0.01(3) |

Å: angstrom; a: a-axis; α: α-angle; b: b-axis; β: β-angle; c: c-axis; CCDC: Cambridge Crystallographic Data Centre; γ: γ-angle; K: Kelvin; R: reliability factor; wR: weighted reliability factor; Z: number of molecules per unit cell.

**Supplementary Table S2.** RG7774 Selectivity for mouse and human endocannabinoid enzymes.

|  | RG7774 10 µM | | Positive control | | |
| --- | --- | --- | --- | --- | --- |
| Assay | Mean ± SEM % inhibition of control | p-value | Positive control [concentration] | Mean ± SEM % inhibition of control | *p* value |
| Mouse CB1R (competitive binding assay) | 3.8 ± 12.5 | NS | Rimonabant [1 µM] | 111.2 ± 5.6 | < 0.005 |
| Mouse CB1R (cAMP assay) | 16.6 ± 23.5 | NS | CP-55940 [0.1 µM] | 91.5 ± 2.6 | < 0.001 |
| Human  5-LOX* | –20.5 ± 3.2 | NS | NDGA [100 µM] | 126.6 ± 2.4 | < 0.005 |
| Human  12-LOX* | 1.5 ± 1.8 | NS | NDGA [100 µM] | 84.6 ± 0.7 | < 0.001 |
| Human  15-LOX* | –8.7 ± 4.0 | NS | NDGA [100 µM] | 112.2 ± 0.9 | < 0.005 |
| Human MAGL* | –9.7 ± 3.6 | NS | JZL-184 [100 nM] | 84.3 ± 3.4 | < 0.05 |
| Human DAGL* | 4.2 ± 52.2 | NS | DO53 [10 µM] | 103.1 ± 13.2 | <0.05 |
| Human* EMT | 4.5 ± 25.5 | NS | OMDM-1 [40 µM] | 95.5 ± 0.4 | < 0.05 |
| Human FAAH* | –5.2 ± 0.6 | NS | URB-597 [100 nM] | 92.8 ± 6.1 | < 0.001 |
| Mouse MAGL | 2.7 ± 11.7 | NS | JLZ-184 [100 nM] | 76.4 ± 5.9 | < 0.05 |
| Mouse DAGL | 17.0 ± 17.7 | NS | DO53 [10 µM] | 60.4 ± 4.3 | < 0.05 |
| Mouse FAAH | 6.0 ± 8.7 | NS | URB-597 [100 nM] | 83.2 ± 4.6 | < 0.05 |

*Similar experiments were carried out using 8 concentrations of RG7774 (0.1–500 μM); RG7774 did not achieve significant levels of CB1R inhibition at any dose. Data reported are the mean (± SEM) of at least 2 independent experiments, each performed in duplicate except for the EMT assay, which was performed in triplicate. Statistical analyses were performed using the nonparametric Mann-Whitney U-test and the Instat3 program (GraphPAD Software for Science). cAMP: cyclic adenosine monophosphate; CB1R: cannabinoid receptor 1, CB2R: cannabinoid receptor 2; DAGL: **diacylglycerol lipase;**EMT: endocannabinoid membrane transporter; FAAH:, fatty acid amide**hydrolase;** LOX: lipoxygenase; **MAGL: monoacylglycerol lipase;**NS: not significant; SEM: standard error of the mean.

**Supplementary Table S3.** RG7774 binding to non-CB1R/CB2R receptors, ion channels, transporters, and enzymes (CEREP, Celle l’Evescault, France; http://www.cerep.fr).

| 1. Receptors | | | | |
| --- | --- | --- | --- | --- |
| Target | Radioligand | Competitor | Reference compound | % inhibition of control SB (n = 2) |
| A1 (h) | [^3^H]-DPCPX | DPCPX | DPCPX | 3.6 |
| A2A (h) | [^3^H]-CGS 21680 | NECA | NECA | 12.4 |
| A3 (h) | [^125^I]-AB-MECA | IB-MECA | IB-MECA | –0.6 |
| Alpha 1 (non-selective) | [^3^H]-prazosin | prazosin | prazosin | 6.9 |
| Alpha 2 (non-selective) | [^3^H]-RX 821002 | (-)epinephrine | yohimbine | –20.9 |
| Beta 1 (h) | [^3^H](-)CGP 12177 | alprenolol | atenolol | –3.1 |
| Beta 2 (h) | [^3^H](-)CGP 12177 | alprenolol | ICI 118551 | –1.3 |
| AT1 (h) | [^125^I]-[Sar^1^,Ile^8^]-AT-II | angiotensin-II | saralasin | –5.6 |
| AT2 (h) | [^125^I]-CGP 42112A | angiotensin-II | angiotensin-II | 3.1 |
| BB (non-selective) | [^125^I]-[Tyr^4^]bombesin | bombesin | bombesin | –9.5 |
| B2 (h) | [^3^H]-bradykinin | bradykinin | NPC 567 | –1.6 |
| CGRP (h) | [^125^I]-hCGRPα | hCGRPα | hCGRPα | 1.7 |
| CB1 (h) | [^3^H]-CP 55940 | WIN 55212-2 | CP 55940 | –0.8 |
| CCK1 (CCKA) (h) | [^125^I]-CCK-8s | CCK-8s | CCK-8s | –7.4 |
| CCK2 (CCKB) (h) | [^125^I]-CCK-8s | CCK-8s | CCK-8s | –7.6 |
| D1 (h) | [^3^H]-SCH 23390 | SCH 23390 | SCH 23390 | –1.0 |
| D2S (h) | [^3^H]-methyl-spiperone | (+)butaclamol | (+)butaclamol | 12.0 |
| D3 (h) | [^3^H]-methyl-spiperone | (+)butaclamol | (+)butaclamol | –0.3 |
| D4.4 (h) | [^3^H]-methyl-spiperone | (+)butaclamol | clozapine | 3.7 |
| D5 | [^3^H]-SCH 23390 | SCH 23390 | SCH 23390 | –2.0 |
| ETA (h) | [^125^I]-endothelin-1 | endothelin-1 | endothelin-1 | –10.4 |
| ETB (h) | [^125^I]-endothelin-1 | endothelin-1 | endothelin-3 | –8.1 |
| GABA (non-selective) | [^3^H]-GABA | GABA | GABA | –16.1 |
| GAL1 | [^125^I]-galanin | galanin | galanin | –9.1 |
| GAL2 | [^125^I]-galanin | galanin | galanin | –4.0 |
| GR (h) | [^3^H]-dexamethasone | dexamethasone | dexamethasone | 3.0 |
| PDGF | [^125^I]-PDGF BB | PDGF BB | PDGF BB | –5.6 |
| CXCR2 | [^125^I]-IL-8 | IL-8 | IL-8 | –7.7 |
| CCR1 | [^125^I]-MIP-1α | MIP-1α | MIP-1α | 5.6 |
| TNF-α | [^125^I]-TNF-α | TNF-α | TNF-α | 4.9 |
| H1 (h) | [^3^H]-pyrilamine | pyrilamine | pyrilamine | –8.8 |
| H2 (h) | [^125^I]-APT | tiotidine | cimetidine | –2.3 |
| MC4 (h) | [^125^I]-NDP-α-MSH | NDP-α-MSH | NDP-α-MSH | –14.5 |
| MT1 (ML1A) (h) | [^125^I]-2-iodomelatonin | melatonin | melatonin | 7.9 |
| M1 (h) | [^3^H]-pirenzepine | atropine | pirenzepine | –15.6 |
| M2 (h) | [^3^H]-AF-DX 384 | atropine | methoctramine | –8.7 |
| M3 (h) | [^3^H]-4-DAMP | atropine | 4-DAMP | –6.7 |
| M4 (h) | [^3^H]-4-DAMP | atropine | 4-DAMP | –8.9 |
| M5 (h) | [^3^H]-4-DAMP | atropine | 4-DAMP | –2.7 |
| NK1 (h) | [^125^I]-BH-SP | [Sar^9^,Met(O2)^11^]-SP | [Sar^9^,Met(O_2_)^11^]-SP | –3.7 |
| NK2 (h) | [^125^I]-NKA | [Nleu^10^]-NKA (4-10) | [Nleu^10^]-NKA (4-10) | –5.5 |
| NK3 (h) | [^3^H]-SR 142801 | SB 222200 | SB 222200 | 8.2 |
| Y1 (h) | [^125^I]-peptide YY | NPY | NPY | –5.5 |
| Y2 (h) | [^125^I]-peptide YY | NPY | NPY | –3.6 |
| NTS1 (NT1) (h) | [^125^I]-Tyr3-neurotensin | neurotesin | neurotesin | –4.7 |
| δ(DOP) (h) | [^3^H]-DADLE | naltrexone | DPDPE | 4.7 |
| κ(KOP) | [^3^H]-U 69593 | naloxone | U 50488 | –12.7 |
| μ(MOP) (h) | [^3^H]-DAMGO | naloxone | DAMGO | –2.5 |
| NOP (ORL1) (h) | [^3^H]-nociceptin | nociceptin | nociceptin | 2.7 |
| PAC1 (PACAP) (h) | [^125^I]-PACAP_1-27_ | [^125^I]PACAP_1-27_ | PACAP_1-38_ | –1.3 |
| PCP | [^3^H]-TCP | MK 801 | MK 801 | –4.4 |
| PPARγ (h) | [^3^H]-rosiglitazone | rosiglitazon | rosiglitazon | 1.7 |
| EP2 (h) | [^3^H]-PGE_2_ | PGE_2_ | PGE_2_ | –13.4 |
| EP4 (h) | [^3^H]-PGE_2_ | PGE_2_ | PGE_2_ | –8.7 |
| IP (PGI2) (h) | [^3^H]-iloprost | iloprost | iloprost | –3.8 |
| P2X | [^3^H]-α,β-MeATP | α,β-MeATP | α,β-MeATP | 25.9 |
| P2Y | [^35^S]-dATPαS | dATPαS | dATPαS | 2.3 |
| 5-HT1A (h) | [^3^H]-8-OH-DPAT | 8-OH-DPAT | 8-OH-DPAT | 3.6 |
| 5-HT1B (h) | [^125^I]-CYP (+ 30 μM isoproterenol) | serotonin | serotonin | –10.6 |
| 5-HT2A (h) | [^3^H]-ketanserin | ketanserin | ketanserin | –4.8 |
| 5-HT2B (h) | [^125^I](±)DOI | (±)DOI | (±)DOI | –24.1 |
| 5-HT2C (h) | [^3^H]-mesulergine | RS 102221 | RS 102221 | 2.7 |
| 5-HT5A (h) | [^3^H]-LSD | serotonin | serotonin | 2.2 |
| 5-HT6 (h) | [^3^H]-LSD | serotonin | serotonin | 3.5 |
| 5-HT7 (h) | [^3^H]-LSD | serotonin | serotonin | 3.9 |
| sigma (non-selective) (h) | [^3^H]-DTG | haloperidol | haloperidol | –8.2 |
| sst (non-selective) | [^125^I]-Tyr^11^-somatostatin-14 | somatostatin-14 | somatostatin-14 | –22.2 |
| VPAC1 (VIP1) (h) | [^125^I]-VIP | VIP | VIP | 7.8 |
| V1a (h) | [^3^H]-AVP | AVP | [d(CH_2_)_5_^1^,Tyr(Me)_2_]-AVP | 7.5 |
| 2. Ion channels | | | | |
| 5-HT3 (h) | [^3^H]-BRL 43694 | MDL 72222 | MDL 72222 | 1.4 |
| Ca2+ channel (L, verapamil site) (phenylalkylamine) | [^3^H]-D888 | D 600 | D 600 | 3.0 |
| BZD (central) | [^3^H]-flunitrazepam | diazepam | diazepam | –0.7 |
| BZD (peripheral) | [^3^H]-PK 11195 | PK 11195 | PK 11195 | –12.0 |
| KV channel | [^125^I]-α-dendrotoxin | α-dendrotoxin | α-dendrotoxin | –5.7 |
| SKCa channel | [^125^I]-apamin | apamin | apamin | 8.6 |
| Cl- channel (GABA-gated) | [^35^S]-TBPS | picrotoxinin | picrotoxinin | 17.2 |
| 3. Transporters | | | | |
| Norepinephrine transporter (h) | [^3^H]-nisoxetine | desipramine | protriptyline | 5.4 |
| Dopamine transporter (h) | [^3^H]-BTCP | BTCP | BTCP | 4.9 |
| 5-HT transporter (h) | [^3^H]-imipramine | imipramine | imipramine | 22.2 |

None of the effects reported in this table were significant.

**References**

1. Kerns EH, Di L, Carter GT. In vitro solubility assays in drug discovery. Curr Drug Metab (2008) 9:879-85. doi: 10.2174/138920008786485100

2. Jakubiak P, Wagner B, Grimm HP, Petrig-Schaffland J, Schuler F, Alvarez-Sanchez R. Development of a Unified Dissolution and Precipitation Model and Its Use for the Prediction of Oral Drug Absorption. Mol Pharm (2016) 13:586-98. doi: 10.1021/acs.molpharmaceut.5b00808

3. Kansy M, Senner F, Gubernator K. Physicochemical high throughput screening: parallel artificial membrane permeation assay in the description of passive absorption processes. J Med Chem (1998) 41:1007-10. doi: 10.1021/jm970530e

4. Di L, Keefer C, Scott DO, Strelevitz TJ, Chang G, Bi YA, et al. Mechanistic insights from comparing intrinsic clearance values between human liver microsomes and hepatocytes to guide drug design. Eur J Med Chem (2012) 57:441-8. doi: 10.1016/j.ejmech.2012.06.043

5. LeCluyse EL, Witek RP, Andersen ME, Powers MJ. Organotypic liver culture models: meeting current challenges in toxicity testing. Crit Rev Toxicol (2012) 42:501-48. doi: 10.3109/10408444.2012.682115

6. Banker MJ, Clark TH, Williams JA. Development and validation of a 96-well equilibrium dialysis apparatus for measuring plasma protein binding. J Pharm Sci (2003) 92:967-74. doi: 10.1002/jps.10332

7. Zamek-Gliszczynski MJ, Ruterbories KJ, Ajamie RT, Wickremsinhe ER, Pothuri L, Rao MV, et al. Validation of 96-well equilibrium dialysis with non-radiolabeled drug for definitive measurement of protein binding and application to clinical development of highly-bound drugs. J Pharm Sci (2011) 100:2498-507. doi: 10.1002/jps.22452

8. Poirier A, Cascais AC, Bader U, Portmann R, Brun ME, Walter I, et al. Calibration of in vitro multidrug resistance protein 1 substrate and inhibition assays as a basis to support the prediction of clinically relevant interactions in vivo. Drug Metab Dispos (2014) 42:1411-22. doi: 10.1124/dmd.114.057943

9. Fowler S, Zhang H. In vitro evaluation of reversible and irreversible cytochrome P450 inhibition: current status on methodologies and their utility for predicting drug-drug interactions. AAPS J (2008) 10:410-24. doi: 10.1208/s12248-008-9042-7

10. Brink A, Fontaine F, Marschmann M, Steinhuber B, Cece EN, Zamora I, et al. Post-acquisition analysis of untargeted accurate mass quadrupole time-of-flight MS(E) data for multiple collision-induced neutral losses and fragment ions of glutathione conjugates. Rapid Commun Mass Spectrom (2014) 28:2695-703. doi: 10.1002/rcm.7062

11. Bari M, Battista N, Fezza F, Finazzi-Agro A, Maccarrone M. Lipid rafts control signaling of type-1 cannabinoid receptors in neuronal cells. Implications for anandamide-induced apoptosis. J Biol Chem (2005) 280:12212-20. doi: 10.1074/jbc.M411642200

12. Fezza F, Oddi S, Di Tommaso M, De Simone C, Rapino C, Pasquariello N, et al. Characterization of biotin-anandamide, a novel tool for the visualization of anandamide accumulation. J Lipid Res (2008) 49:1216-23. doi: 10.1194/jlr.M700486-JLR200

13. Gattinoni S, De Simone C, Dallavalle S, Fezza F, Nannei R, Amadio D, et al. Enol carbamates as inhibitors of fatty acid amide hydrolase (FAAH) endowed with high selectivity for FAAH over the other targets of the endocannabinoid system. ChemMedChem (2010) 5:357-60. doi: 10.1002/cmdc.200900472
